# MiR-4327 targets TP53 to promote cervical cancer cell proliferation

**DOI:** 10.1007/s10142-026-01934-9

**Published:** 2026-07-11

**Authors:** Mei-Lin Chen, Chen-Yang Chu, Feng-Xian Zhang, Li-Pei Zhang, Ying Yang, Bai-Zhao Peng, Chuang-Hai Wu, Xiao-Mei Chen, Yan-Ting You, Bing-Ying Rong, Hiu Yee Kwan, Xiao-Shan Zhao, Yan-Yan Liu

**Affiliations:** 1 School of Traditional Chinese Medicine, Southern Medical University, No.1023, South Shatai Road, Baiyun District, Guangzhou, Guangdong 510515 China; 2 School of Chinese Medicine, Hong Kong Baptist University, Hong Kong, 999077 China; 3 Guangdong Basic Research Center of Excellence for Integrated Traditional and Western Medicine for Qingzhi Diseases, Guangzhou, 510515 China

**Keywords:** MiR-4327, Cervical cancer, TP53, Proliferation, Cell cycle

## Abstract

Cervical cancer is a leading cause of female malignancy worldwide. While microRNA-4327 (miR-4327) has been implicated as a potential oncogene, its functional role and molecular mechanisms in cervical cancer pathogenesis remain unclear. This study aimed to determine the oncogenic function of miR-4327 and elucidate its downstream regulatory mechanism in cervical cancer pathogenesis. We measured miR-4327 expression in clinical cervical cancer tissues and cell lines using quantitative real-time PCR (qRT-PCR). We then assessed its effects on proliferation, migration, invasion, and cell cycle progression through functional assays including cell counting kit-8 (CCK-8), colony formation, transwell, and flow cytometry. To evaluate tumor growth in vivo, we established a xenograft model in non-obese diabetic (NOD)-severe combined immune-deficient (scid) mice. Using bioinformatic analysis and luciferase reporter assays, we identified TP53 as a direct target of miR-4327 and further validated this regulatory relationship with gain- and loss-of-function experiments. We found that miR-4327 was significantly upregulated in cervical cancer and promoted malignant phenotypes such as proliferation, migration, invasion, and cell cycle progression in vitro. Consistent with this, overexpression of miR-4327 accelerated tumor growth in vivo. Mechanistically, we confirmed TP53 as a direct functional target of miR-4327. Knocking down TP53 phenocopied the oncogenic effects of miR-4327, while restoring TP53 expression rescued the tumor-promoting effects mediated by miR-4327. These findings reveal a novel miR-4327/TP53 regulatory axis and nominate miR-4327 as a potential therapeutic target for intervention.

**Supplementary Information:**

The online version contains supplementary material available at 10.1007/s10142-026-01934-9.

## Introduction

Cervical cancer is a common gynecological malignant tumor and the fourth most common cancer in women worldwide, following breast cancer, colorectal cancer and lung cancer (Bray et al. 2022). Screening and early diagnosis of cervical cancer are crucial (Harris 2024; Simms et al. 2023). Current screening methods, such as HPV detection and cytology detection, can effectively identify high-risk groups; however, there is a certain rate of misdiagnosis and missed diagnosis (Voelker 2023; Fisher et al. 2024). In the treatment of cervical cancer, surgery and radiotherapy are the primary methods, supplemented by chemotherapy, targeted therapy and immunotherapy (Francoeur et al. 2025). Despite continuous advancements in cancer treatment interventions, the management of advanced and recurrent cervical cancer remains a significant challenge (Francoeur et al. 2025). Moving forward, it is crucial to optimize combination therapies, validate predictive biomarkers, and ensure that innovative treatments are accessible to all populations.

MiRNAs, typically 22 nucleotides in length (Lu and Rothenberg 2018), regulate key cellular processes such as differentiation (Shenoy and Blelloch 2014), proliferation (Schober et al. 2014), and survival (Bagnoli et al. 2016) by binding to target RNAs and inhibiting or degrading mRNA translation (Huntzinger and Izaurralde 2011). In cervical cancer, abnormal miRNA expression is closely associated with tumor spread, invasion, progression, and metastasis, positioning miRNAs as potential biomarkers for the disease (Wang et al. 2014; Li et al. 2011). MiR-4327, a recently identified miRNA, has shown altered expression in several cancers, including lung and esophageal, suggesting its promise as a diagnostic or prognostic marker (Chakraborty and Nath 2022; Okuda et al. 2021). However, the role and expression of miR-4327 in cervical cancer remain underexplored, hindering a complete understanding of its contribution to the disease’s pathogenesis. To investigate the expression pattern and biological function of miR-4327 in cervical cancer and evaluate its potential as a biomarker for early diagnosis or prognosis.

## Material and methods

### Clinical information collection

Clinical data were collected from five cervical cancer patients who underwent surgical treatment at the Southern Medical University Affiliated Hospital of Integrated Traditional Chinese and Western Medicine between January and December 2024. Inclusion criteria were as follows: 1) absence of chronic cardiovascular, pulmonary, hepatic, or renal diseases, with accurate and complete clinical records; 2) no prior chemotherapy before surgery; 3) the study participants provided informed consent. Exclusion criteria included: 1) patients who had undergone hysterectomy; 2) patients with cancers of other organs (including breast, ovarian, gastric, colorectal, or urinary system cancers); 3) patients with severe cardiac, pulmonary, hepatic, or renal dysfunction.

### Cell culture

Human cervical cancer cell lines, HeLa and SiHa, were obtained from Cellcook (CC1101, CC1102, Guangzhou, China) and cultured in Dulbecco’s Modified Eagle Medium (DMEM) supplemented with 10% fetal bovine serum (BI, Israel) and 1% streptomycin-penicillin (ST488, Beyotime, Shanghai, China). Cells were maintained in a humidified incubator at 37 °C with 5% CO₂. HeLa and SiHa cells were passed or cultured regularly and inoculated into different culture plates based on the requirements of each experiment.

### Cell proliferation assays

HeLa or SiHa cells (5 × 10^3^) were seeded in 96-well plates and cultured to 80% confluence. Transfection was performed with Lipo6000™ reagent (C0526, Beyotime, Shanghai, China) using miR-4327 mimics/NC plasmid (100 ng), miR-4327 inhibitor/NC (200 ng), NC-siRNA/TP53 siRNA (200 ng), or vector/TP53 expression vector (100 ng). The miR-4327 plasmids were constructed by RiboBio, and TP53 plasmids by iGeneBIO. After 6 h of miRNA transfection, the medium was replaced with DMEM, and after 4 h of TP53 plasmid transfection, the medium was also replaced. Cell viability was assessed by adding 10 μl CCK8 solution, incubating at 37 °C for 2 h. Finally, the optical density was measured at 450 nm using a microplate reader (Synergy H1, BioTek, Vermont, USA).

### 5-ethynyl-2’-deoxyuridine (EdU) assay

After 24 h of transfection, 5 × 10^3^ cells were plated in 96-well plates and incubated with 100 μl of EdU working solution (KGA331, Beyotime, Shanghai, China) for 3 h at 37°C. Following fixation with 4% paraformaldehyde (PFA) for 30 min, cells were permeabilized with 0.5% Triton X-100 and stained with EdU and DAPI to visualize cell proliferation and DNA synthesis. Fluorescence images were captured using a microscope and analyzed with ImageJ to evaluate EdU incorporation.

### Wound healing assay

To assess the migration potential of cells transfected with different miR-4327 plasmids, 5 × 10^5^ HeLa and SiHa cells were cultured in 6-well plates for 24 h. A sterile pipette tip was used to create a scratch on the monolayer of both cell types. Afterward, the cells were washed three times with PBS to remove detached cells. Following the designated treatment period, the wound area was examined, and images were captured using a phase contrast microscope. The ImageJ analysis tool was employed to calculate the percentage of wound closure and the healed area at 0 and 24 h.

### Transwell migration and invasion assay

Matrigel solution was prepared in pre-cooled medium with 1: 10. After mixing, slowly add to the upper chamber at a rate of 50 μl per well, and incubate in the incubator for about 4 h to solidiate the matrigel solution. The transfected cells were spread in the transwell chamber at 5 × 10^4^, and the cell suspension was prepared with the basic medium. The cells were starved and inoculated in the upper chamber of the transwell chamber with 200 μl per well. The culture medium containing 10% fetal bovine serum was added to the lower chamber, and 500 μl was added to each well. After incubation for 24 h, the invaded cells were fixed and stained with 4% PFA, and the number of invaded cells was recorded under the microscope.

### Clonogenic assay

HeLa and SiHa cells were transfected with different miR-4327 plasmids and seeded in 6-well plates at a density of 1,000 cells per 2 ml. The cells were incubated for 10 days in a cell culture incubator. Upon colony formation, the medium was discarded, and the cells were washed twice with PBS. The cells were then fixed with 4% PFA for 15 min, followed by two additional PBS washes. Subsequently, the cells were stained with crystal violet for 10 min, washed with deionized water, and photographed. Colonies containing more than 50 cells were counted under an optical microscope, and the colony formation rate was calculated using ImageJ software.

### Flow cytometry (FCM)

Cell cycle progression was analyzed by propidium iodide staining. HeLa or SiHa cells were transfected with miR-4327 plasmids for 24 h, then fixed in 70% ethanol and washed with PBS. Following this, cell cycle distribution was assessed using a cell cycle detection kit (Keygen Biotech, KGA511, Jiangsu, China) and red fluorescence signals were detected by flow cytometry (CytoFLEX, Beckman, Brea, USA) at 488 nm. Data were analyzed with FlowJo v10 software.

### Western blot (WB) analysis

Cells were lysed in cell lysis buffer (CW2333S, Cwbio, Beijing, China) with protease and phosphatase inhibitors (BL615A, BL612A, Biosharp, Hefei, China). Protein concentration was measured using a BCA assay kit (23227, Thermo Fisher, Massachusetts, USA). Equal amounts (20 μg) of protein were loaded onto a 12% SDS-PAGE gel, transferred to PVDF membranes, and blocked with 5% BSA. Membranes were incubated with primary antibodies overnight at 4 °C, followed by a 2-h incubation with secondary antibodies at 4°C. Primary antibodies used included anti-N-cadherin (Ncad, 1:1000, 1311S), anti-Cyclin D1 (1:1000, 26939–1-AP), anti-CDK4 (1:1000, 11026–1-AP), anti-TP53 (1:1000, AF0879), and anti-GAPDH (1:10000, AF7021). Ncad and the secondary antibody were purchased from Cell Signaling Technology, while the remaining antibodies were sourced from Proteintech and Affinity. Protein bands were visualized using an ECL kit (WBULS0500, Millipore, Massachusetts, USA) and analyzed with the FluorChem ETM system (5200S, Tanon, Shanghai, China). Band intensities were quantified using ImageJ software.

### Xenograft mouse model

The four-week-old female NOD scid mice (weight about 18-20g) were purchased from Charles River (Beijing, China) and raised in a specific pathogen free environment with free access to water and food. Prior to the experiment, the mice were acclimatized for one week to ensure they could adapt to the experimental environment. Subsequently, the mice were randomly assigned into four groups based on their body weight, with each group consisting of four mice. HeLa cells (5 × 10^6^ cells) transfected with different miR-4327 plasmids were subcutaneously injected into the right axilla of each mouse for subsequent experimental treatment and observation. The groups were as follows: overexpression control group (transfected with mimics NC), miRNA overexpression group (transfected with miR-4327 mimics), inhibition control group (transfected with inhibitor NC), and miRNA inhibition group (transfected with miR-4327 inhibitor). Tumor size was measured every three days using specialized calipers to monitor tumor growth. Tumor volume = 0.5 × length × width^2^. After 14 days of culture and observation, all mice were sacrificed, and tumor tissues were collected for further analysis. All animal experiments were performed with four mice per experimental group (*n* = 4).

### Transcriptomic analysis

Human cervical cancer HeLa or SiHa cells were transfected with miR-4327 mimics for 24 h at 37°C. Cells transfected with miR-4327 NC served as the control group. Total RNA was extracted from the cervical cancer cells using Trizol reagent. The PCR-enriched cDNA library was then subjected to sequencing on the Illumina HiSeq high-throughput sequencing platform. Raw data underwent quality control using fastp. Differential expression analysis was performed using the Limma package, with a threshold set at *p* < 0.05. Gene enrichment analysis was conducted using the online platform, the Database for Annotation, Visualization, and Integrated Discovery (DAVID) (https://david.ncifcrf.gov).

### Quantitative real-time PCR (RT-qPCR)

Total RNA was extracted from human CC tissue by Trizol reagent. We used the Evo M-MLV Reverse Transcription kit (AG11711, Accurate Biology, Hunan, China) transcribed cDNA and determined miR-4327 expression and mRNA expression of target gene with SYBR Green Pro Taq HS kit (AG11701, Accurate Biology, Hunan, China). The data were normalized to RNU6B (U6) gene expression. The primer sequence is as follows:

TP53:

- Forward: CCGGACGATATTGAACAATG
- Reverse: CAAGAAGCCCAGACGGAAAC

### Luciferase reporter assay

The luciferase reporter gene assay is a powerful tool for monitoring the interaction between microRNAs and their predicted target mRNAs (Bartel 2009; Gu et al. 2009). HEK-293T cells were co-transfected with the luciferase reporter plasmid pEZX-MT06 (wild-type or mutant TP53 3’ UTR) and miR-4327 mimic using Lipofectamine 6000. After 24 h, the medium was replaced with fresh DMEM containing 10% fetal bovine serum. Luciferase activity was measured using a dual-luciferase assay (GeneCopoeia, LF031, Guangzhou, China) and quantified with a microplate reader. Secreted alkaline phosphatase luminescence was used for transfection efficiency normalization.

### Histological analysis

Tumor tissues were fixed in 4% PFA, embedded in paraffin, and sectioned into 4 μm slices. Sections were stained with hematoxylin and eosin (H&E). For immunohistochemistry (IHC) staining, tissue sections are incubated with anti-Ki 67 (1:200, AF0198, Affinity, Shanghai, China) or anti-TP53 (1:200) overnight. After secondary antibody incubation for 30 min, images were captured at × 200 magnification (scale bar = 100 μm).

### Statistical analysis

All data visualizations and statistical analyses were conducted using GraphPad Prism 8.0 (San Diego, USA). Results are presented as mean ± standard deviation. Comparisons between two groups were made using an unpaired Student's t-test. For comparisons involving more than two groups, one-way ANOVA followed by Tukey's multiple comparisons test was applied. Both one-way and two-way ANOVA were employed as appropriate. All experiments were repeated at least three times, and representative data are shown. A *p*-value of < 0.05 was considered statistically significant.

## Results

### MiR-4327 is significantly upregulated in human cervical cancer tissues

**Fig. 1:**
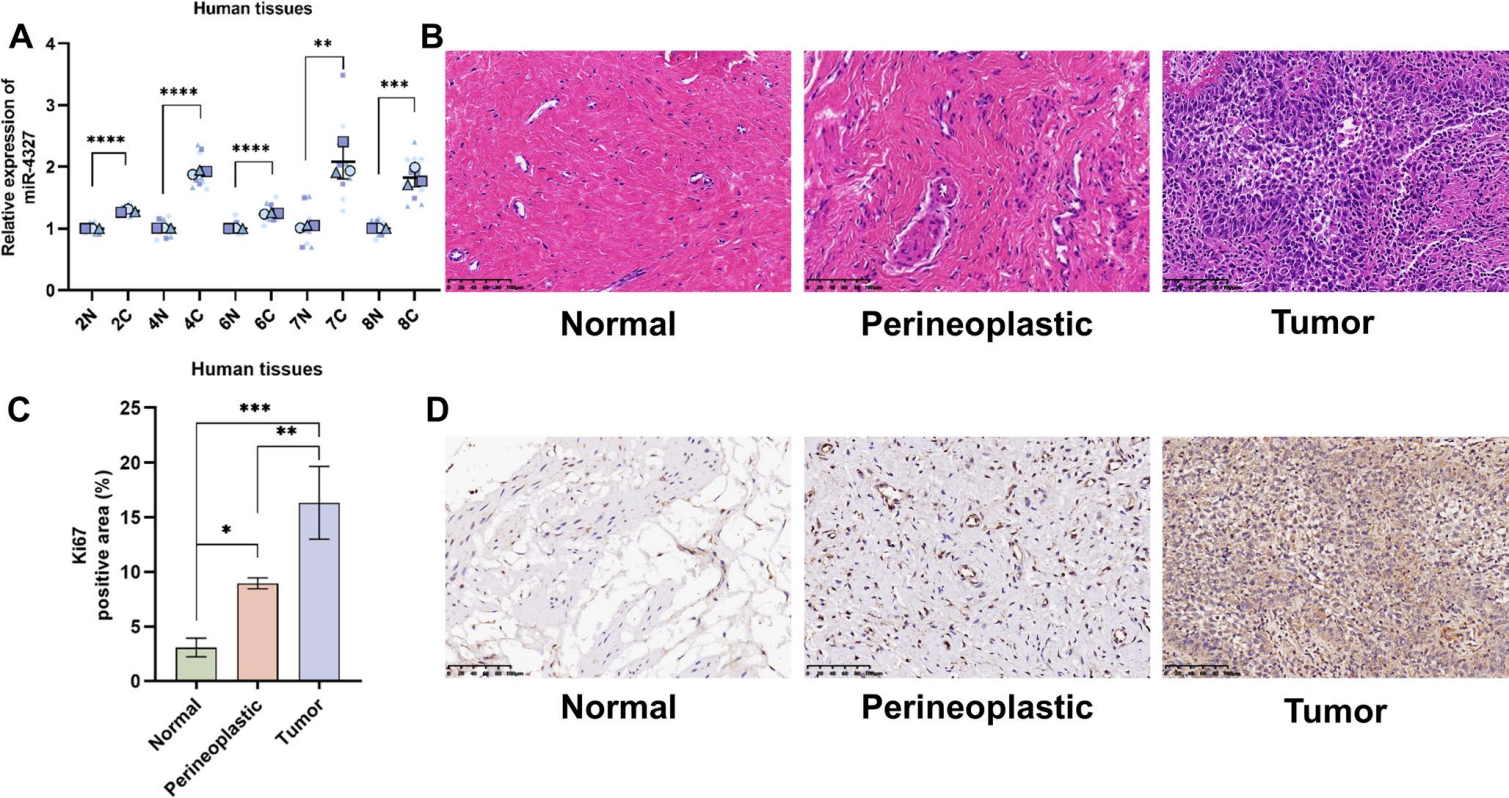
MiR-4327 is signifcantly upregulated in human cervical cancer tissues. (**A**) The expression of miR-4327 in cervical cancer tissues. (**B**) H&E staining of normal cervix, perineo plastic and tumor tissues. Scale bars show 100 μm. (**C** and **D**) Analysis of Ki67 IHC staining of cervical tissues. (**C**) Quantitative analysis of Ki67 positive cells. (**D**) Representative IHC staining results of normal cervical tissues, paracancerous tissues and cervical cancer tissues. Scale bars show 100 μm. Normal: normal cervical tissues, CC: cervical cancer tissue, Perineoplastic: paracancerous tissues, Tumor: cervical cancer tissues. *p < 0.05, **p < 0.01, ***p < 0.001 and ***.*p < 0.0001

In this study, five pairs of cervical cancer tissues and their adjacent normal tissue samples were selected for analysis. MiR-4327 expression was significantly higher in cervical cancer tissues compared to normal tissues (Fig. 1A). Pathological analysis via H&E staining revealed that normal tissues exhibited fewer tumor cells, mild inflammatory response, and low mitotic activity, while adjacent tissues showed disordered cell arrangement and abnormal proliferation. In contrast, tumor tissues had increased cell count, prominent cellular atypia, and frequent mitosis (Fig. 1B). Additionally, IHC analysis of Ki67 expression in cervical tissues showed varying Ki67 positivity across different tissue stages (Fig. 1C and D). Notably, the proportion of Ki67-positive cells was significantly higher in cancer tissues compared to both normal and adjacent tissues. Taken together, these molecular and morphological findings suggest that miR-4327 may play a crucial role in the initiation and progression of cervical cancer, potentially by promoting cell proliferation.

### MiR-4327 promotes proliferation, migration, invasion and progression of cervical cancer cells and induces cell cycle transition

**Fig. 2:**
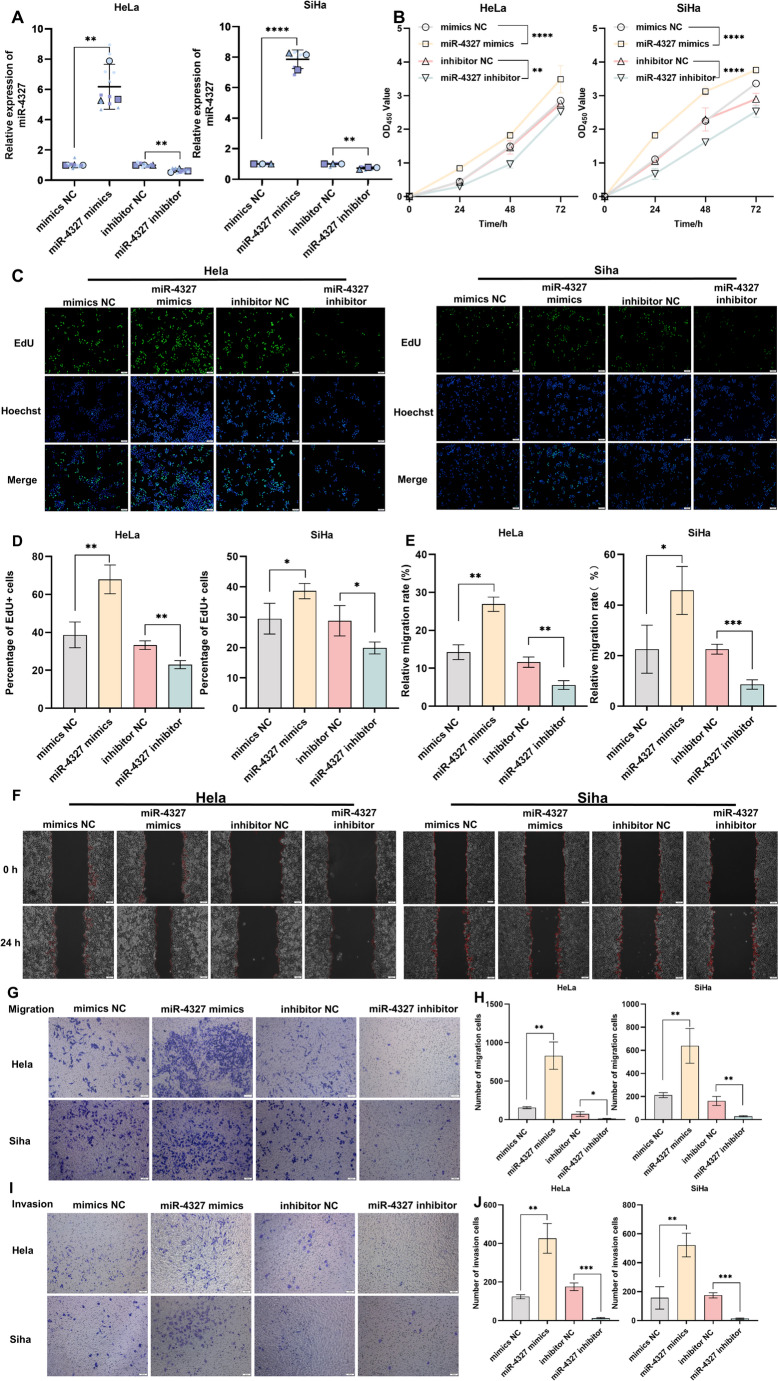
MiR-4327 promotes proliferation, migration, invasion and progression of cervical cancer cells and induces cell cycle transition. (**A**) Expression of miR-4327 after plasmid transfection in HeLa and SiHa cells. (**B**) Effect of miR-4327 on the activity of cervical cancer cells detected by CCK8 assay. (**C**) EdU assay to detect the proliferative ability of miR-4327 regulating cervical cancer cells. Scale bars show 100 μm. (**D**) Quantitative results of EdU assay of HeLa and SiHa cells. (**E** and **F**) Wound healing assay to analyze the regulation of miR-4327 on the migration ability of cervical cancer cells. (**E**) HeLa and SiHa cells migration quantification. (**F**) HeLa and SiHa cell wound healing process. Scale bars show 100 μm. (**G**-**J**) Transwell assay to analyze the regulation of miR-4327 on the migration and invasion ability of cervical cancer cells. (**G**) Representative images of cell migration. Scale bars show 100 μm. (**H**) Quantitative analysis of migration of HeLa and SiHa cells. (**I**) Representative images of cell invasion. Scale bars show 100 μm. (**J**) Quantitative analysis of invasion of HeLa and SiHa cells. ns, no significance, ^*^p < 0.05, ^**^p < 0.01, ^***^p < 0.001, ^****^p < 0.0001

To investigate the role of miR-4327 in cervical cancer cells, miR-4327 mimics or inhibitor plasmids were transfected into HeLa and SiHa cells, and transfection efficiency was confirmed by RT-qPCR (Fig. 2A). To assess the impact of miR-4327 on cervical cancer cell proliferation, cell viability was measured over three days using the CCK8 assay (Fig. 2B). Additionally, the kFluor488-EdU cell proliferation assay was performed to further evaluate the effects of miR-4327 on cell proliferation (Fig. 2C and D). The results revealed that overexpression of miR-4327 significantly enhanced cell proliferation, while inhibition of miR-4327 reduced it. A scratch wound-healing assay revealed that miR-4327 overexpression accelerated wound healing, while inhibition slowed it (Fig. 2E and F). Furthermore, Transwell chamber assays were performed to evaluate cell migration and invasion (Fig. 2G and J). The findings indicated that overexpression of miR-4327 in HeLa and SiHa cervical cancer cells led to a significant increase in the number of cells migrating through the chambers compared to the miR-4327 mimics NC group. In contrast, the number of cells passing through the chambers was significantly reduced in the miR-4327 inhibitor group compared to the inhibitor NC group.

**Fig. 3:**
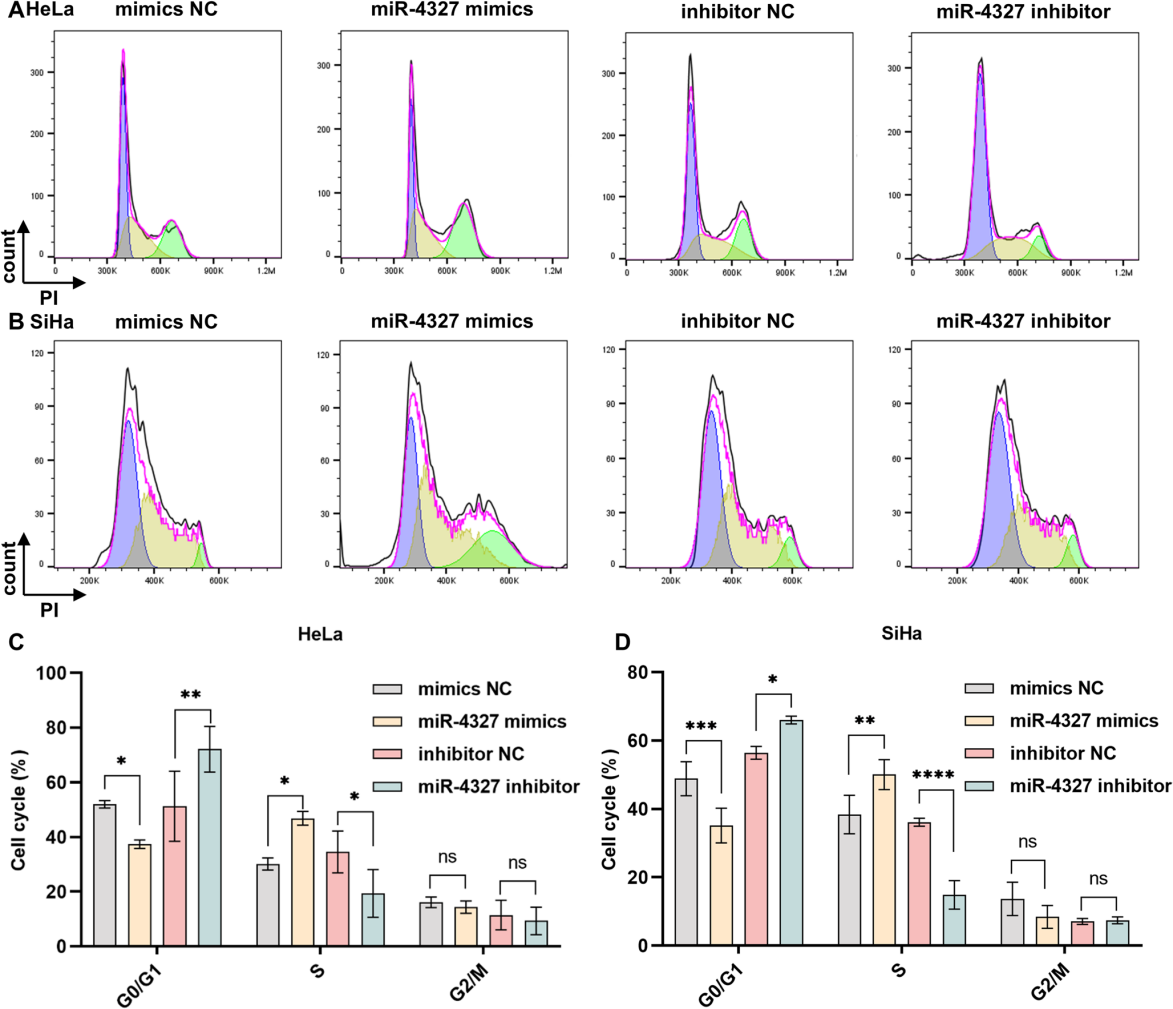
MiR-4327 regulates the cell cycle distribution of cervical cells by flow cytometry. (**A** and **B**) The cell cycle changes of HeLa and SiHa transfected with miR-4327 plasmid for 24 h were detected by flow cytometry. (**C** and **D**) Quantitative analysis of the proportion of cells at each stage of the HeLa and SiHa cell cycle. ns, no significance, *p < 0.05, **p < 0.01, ***p < 0.001, ****p < 0.0001

To further explore the mechanism underlying miR-4327 promotion of proliferation, we analyzed its effect on the cell cycle of cervical cancer cells (Fig. 3A and B). The data showed that in the miR-4327 mimics group, the proportion of cells in the G1 phase decreased compared to the mimics NC group, whereas the proportion of cells in the G1 phase increased in the miR-4327 inhibitor group compared to the inhibitor NC group (Fig. 3C and D).

### MiR-4327 increases the tumorigenicity of HeLa cells in the NOD scid mouse model

**Fig. 4:**
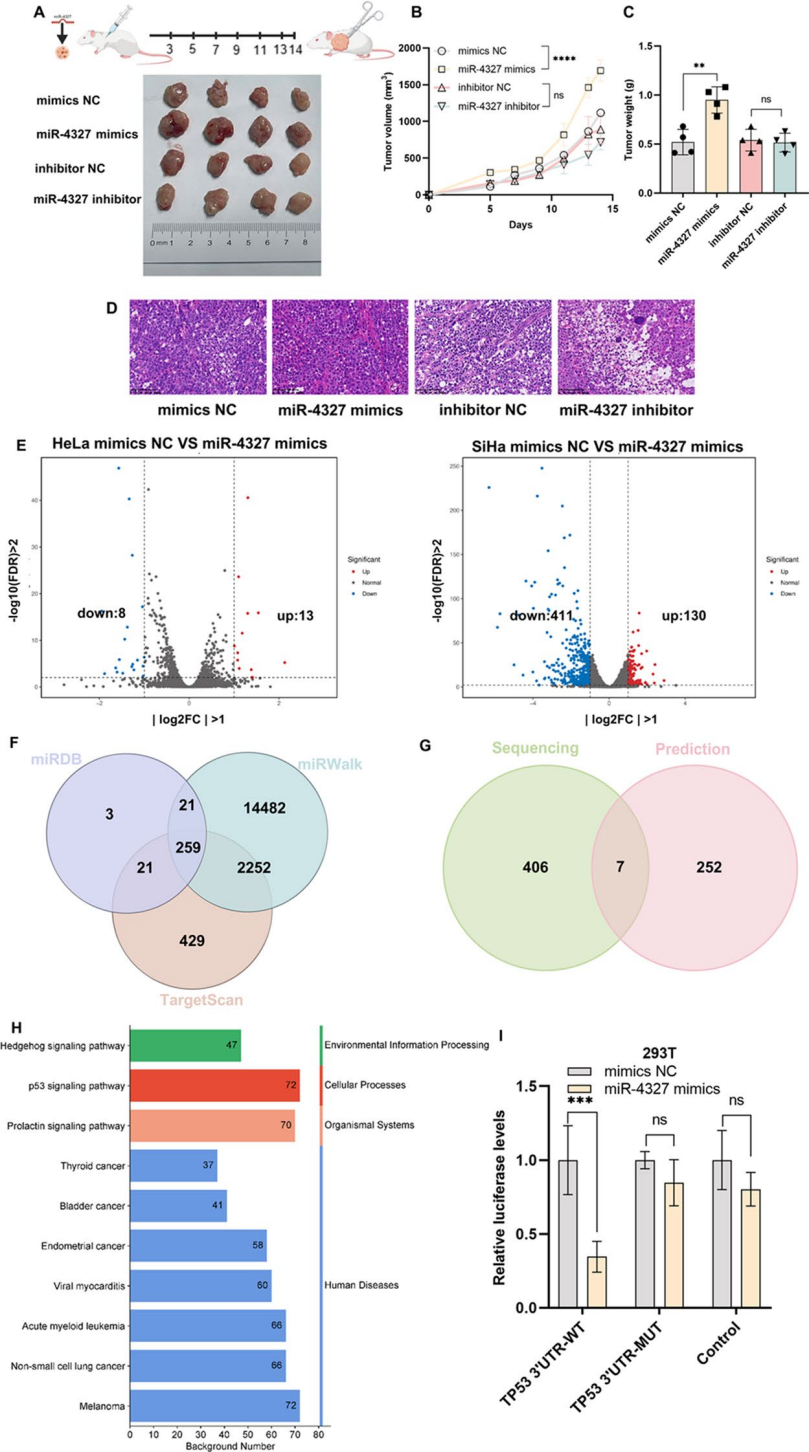
MiR-4327 drives cervical cancer tumorigenesis through transcriptional suppression of TP53. (**A**) Subcutaneous xenograft tumor model was established in NOD scid mice by transplanting HeLa cells transfected with miR-4327 plasmids(n = 4 per group). Tumor length and width were measured every other day to calculate tumor volume. On day14 post-inoculation, the animals were euthanized, and tumor tissues were harvested for further analysis. Anatomical visualization of transplanted tumors, n = 4. (**B**) Dynamic change curve of tumor volume. *p* < 0.0001 for mimics NC vs. miR-4327 mimics; p = 0.0694 for inhibitor NC vs. miR-4327 inhibitor. (**C**) Statistical analysis of tumor weight. *p* = 0.0012 for mimics NC vs. miR-4327 mimics; *p* = 0.9903 for inhibitor NC vs. miR-4327 inhibitor. (**D**) H&E staining showed histopathology changes in the xenograft. Scale bar showing 100μm. (E) Transcriptome sequencing differentially expressed volcano map. (**F**) Multi-database prediction of target genes Venn diagram. (**G**) MiR-4327 transcriptomics sequencing differentially expressed genes and database prediction of target genes intersecting Venn diagram. (**H**) Intersecting target genes KEGG pathway enrichment analysis map. (**I**) Dual luciferase reporter gene validation of miR-4327targeting to TP53-3’ UTR. ns, no significance, **p < 0.01,***p < 0.001and.****p < 0.0001

To investigate the impact of miR-4327 on the tumorigenic potential of cervical cancer cells in vivo, HeLa cells transfected with miR-4327 were subcutaneously inoculated into the right axilla of immunodeficient mice, with 5 × 10^6^ cells injected per mouse. Each experimental group contained 4 mice (*n* = 4 per group). The mice’s body weight, along with the length and width of the tumors, were measured every three days to calculate tumor volume. On day 14 post-intervention, the mice were sacrificed to assess tumor morphology (Fig. 4A). The tumor volume in the miR-4327 mimics group was significantly larger than that in the mimics NC group (*p* < 0.0001), whereas no significant difference was observed between the inhibitor NC and miR-4327 inhibitor groups (*p* = 0.0694) (Fig. 4B). Tumor dissection confirmed increased tumor volume in the mimics group, while the difference in tumor volume and weight between the miR-4327 inhibitor and inhibitor NC groups was not statistically significant (Fig. 4C). For final tumor weight, the miR-4327 mimics group had significantly higher weight compared with the mimics NC group (*p* = 0.0012), while no significant difference was detected between the inhibitor NC and miR-4327 inhibitor groups (*p* = 0.9903)**.** These results suggest that miR-4327 enhances the tumorigenic potential of HeLa cells. H&E staining revealed that tumors in the miR-4327 mimics group had densely packed cells with varying nuclear sizes, hyper staining, higher cell density, and pronounced cell atypia, while the miR-4327 inhibitor group exhibited significant apoptosis, necrosis, and reduced inflammatory cell infiltration (Fig. 4D).

### Pathway enrichment analysis and target gene prediction

To investigate the molecular mechanism by which miR-4327 regulates cervical cancer progression, HeLa and SiHa cells were transfected with miR-4327 mimics or mimics NC, followed by transcriptome sequencing. Based on a screening criterion of |log2FC|> 1, we identified 413 differentially expressed genes (Fig. 4E). To precisely pinpoint the direct targets of miR-4327, we utilized bioinformatics prediction tools, including miRDB, miRwalk, and TargetScan, which predicted 259 potential target genes for miR-4327 (Fig. 4F). We then intersected the differentially expressed genes from the transcriptomic data with the predicted target genes (Fig. 4G), followed by KEGG pathway enrichment analysis of the overlapping genes, which revealed significant enrichment in the p53 signaling pathway (Fig. 4H). TP53 is often referred to as the “guardian of the genome” due to its role in the initiation and progression of various cancers, including cervical cancer. We hypothesize that miR-4327 promotes cancer cell proliferation via the p53 pathway, particularly by modulating the TP53 gene.

To confirm TP53 as a direct target of miR-4327, a dual-luciferase reporter assay was performed (Fig. 4I). HEK-293 T cells were co-transfected with miR-4327 mimics or mimics NC, along with either WT or MUT TP53-3’UTR reporter vectors. The results showed a significant reduction in luciferase activity in the miR-4327 mimics + WT TP53-3’UTR group compared to the mimics NC group, while no change was observed in the miR-4327 mimics + MUT TP53-3’UTR group. This suggests that miR-4327 specifically targets the TP53-3’UTR, regulating TP53 expression. These findings provide experimental evidence for miR-4327’s potential role in cervical cancer.

### Decreased expression of TP53 promotes proliferation of cervical cancer cells

**Fig. 5:**
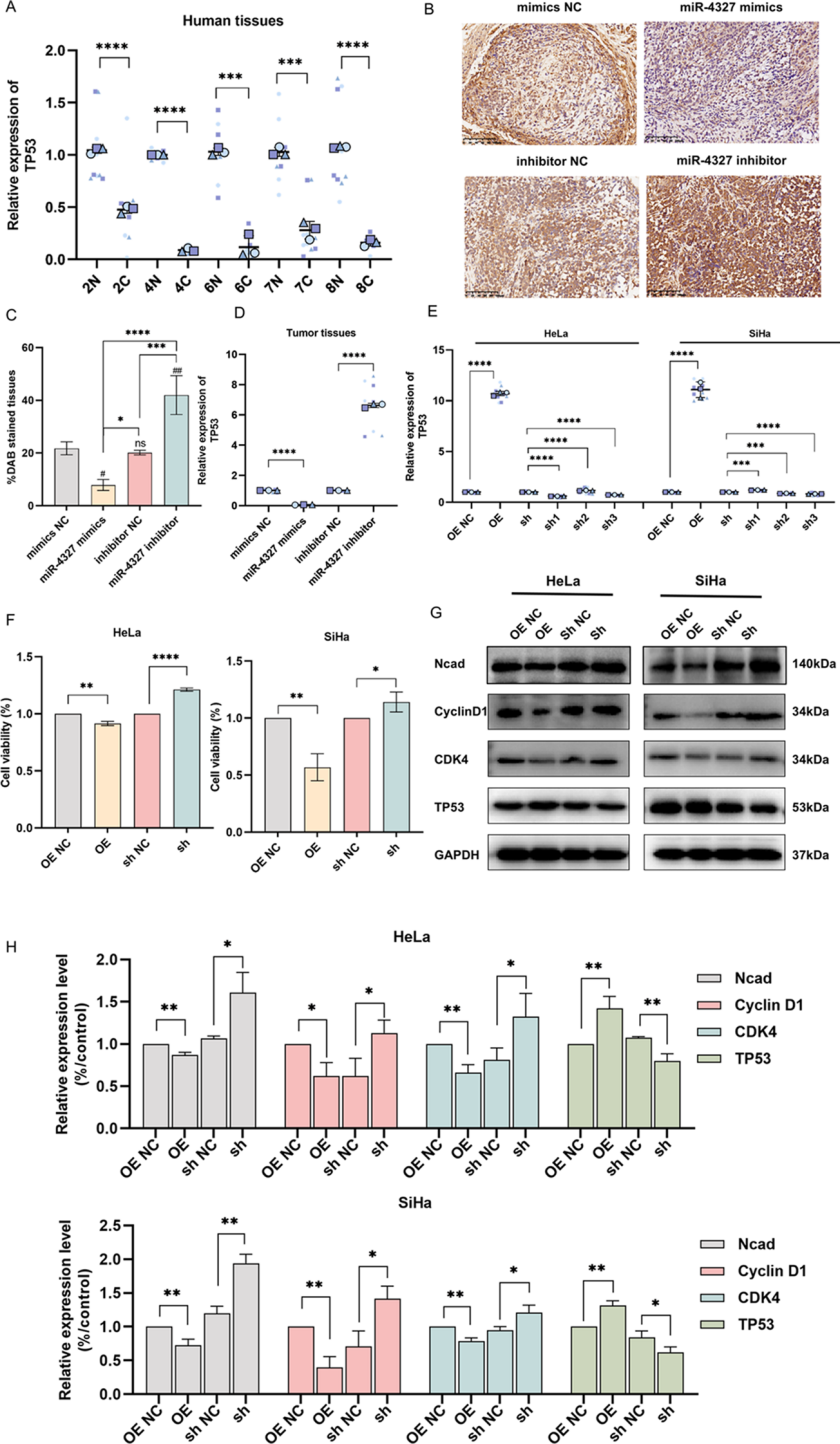
Decreased expression of TP53 promotes proliferation of cervical cancer cells. (**A**) Expression level of TP53 mRNA in cervical cancer tissues detected by RT-qPCR. (**B**-**D**) Characterization of TP53 expression in cervical cancer transplant tumors. (**B**) Representative IHC staining results. Scale bar showing 100 μm. (**C**) Quantitative analysis histogram. (**D**) Detection of TP53 mRNA expression level in mice transplantation tumor tissues by RT-qPCR. (**E**-**G**) Validation of TP53 gene overexpression and knockdown efficiency. #*p* <0.05, ##*p* <0.01, compared with sh NC group. (**E**) Detection of TP53 mRNA expression levels in HeLa and SiHa cells by RT-qPCR. (**F**) Changes in viability of HeLa and SiHa cells after over-expression and knockdown of TP53 gene. (**G**-**H**) Expression analysis of proliferation and cell cycle related proteins in cervical cancer cells regulated by TP53 gene. (**G**) Changes in the levels of proliferation and cell cycle-related proteins in cells after overexpression or knockdown of TP53. (**H**) Histogram of statistical analysis of data on proliferation and cell cycle protein levels in HeLa and SiHa cells. *p <0.05, **p <0.01, ***p <0.001, ****p <0.0001. #*p* <0.05, ##*p* <0.01, compared with mimics NC group

To verify TP53 as a target of miR-4327, we analyzed TP53 mRNA expression in cervical cancer and adjacent normal tissues using RT-qPCR (Fig. 5A). TP53 mRNA was significantly lower in cancer tissues. IHC of tumor tissues from animal models showed reduced TP53 expression in miR-4327-overexpressing tumors, and increased expression when miR-4327 was inhibited (Fig. 5B and C). Additionally, RT-qPCR analysis confirmed that TP53 expression was significantly reduced in the miR-4327 mimics group compared to the mimics NC group (Fig. 5D). To elucidate the role of TP53 in cervical cancer cell proliferation, we conducted TP53 overexpression and knockdown experiments in HeLa and SiHa cell lines, respectively (Fig. 5E). TP53 overexpression and knockdown in HeLa and SiHa cells demonstrated that TP53 overexpression inhibited cell activity, while knockdown enhanced it, similar to miR-4327 overexpression (Fig. 5F). WB analysis showed that TP53 knockdown upregulated Ncad, promoting epithelial-to-mesenchymal transition, while Cyclin D1 and CDK4 upregulation led to abnormal cell cycle progression (Fig. 5G and H). These findings suggest that TP53, regulated by miR-4327, plays a critical role in promoting the proliferation and metastasis of cervical cancer cells.

### Overexpression of TP53 restores the inhibitory effect of miR-4327 on cervical cancer

**Fig. 6:**
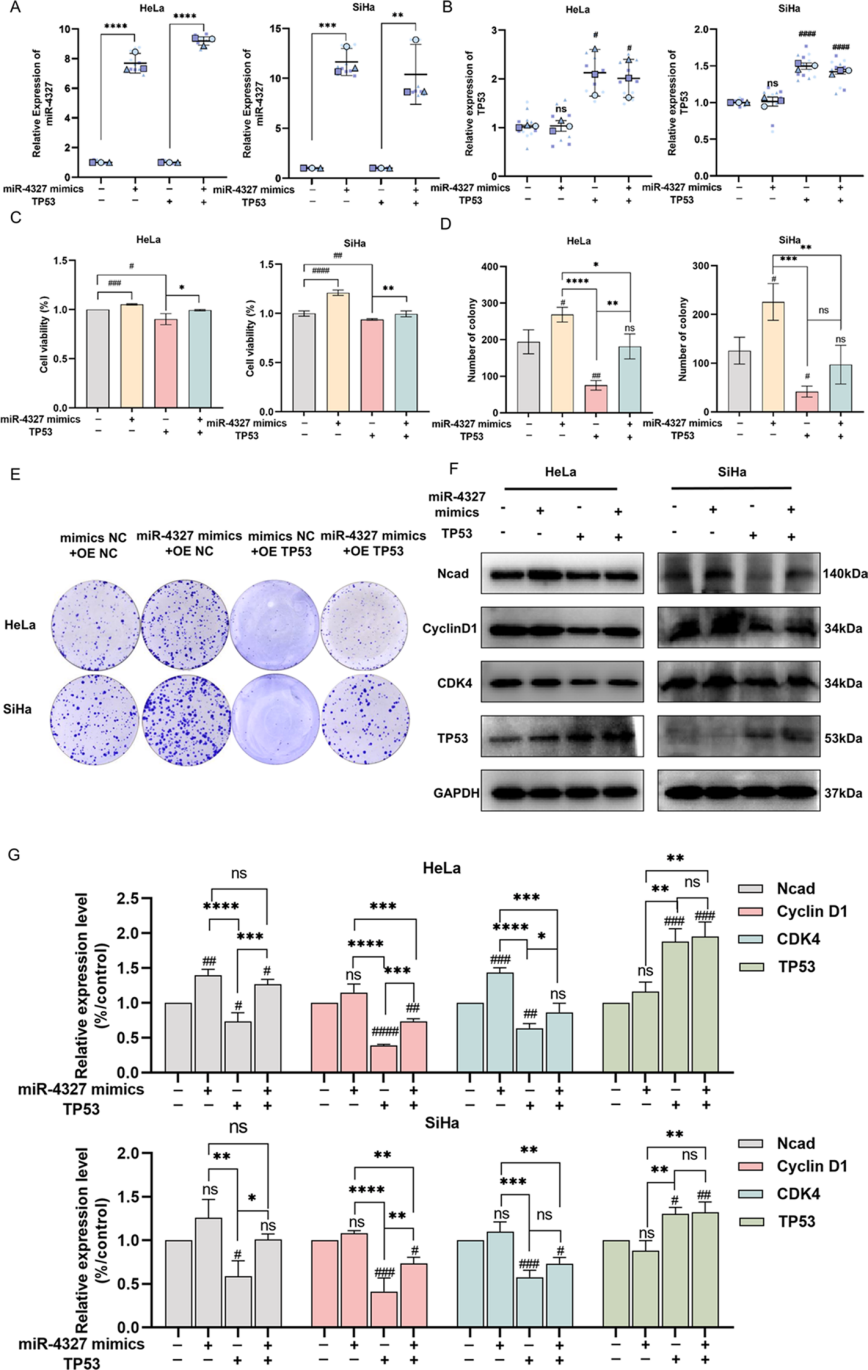
Overexpression of TP53 restores the inhibitory effect of miR-4327 on cervical cancer. (**A**-**B**) The expression of TP53 in cervical cells transfected with plasmid by RT-qPCR. (**A**) Detection of miR-4327 expression levels in HeLa and SiHa cells by RT-qPCR. (**B**) Detection of TP53 mRNA expression levels in HeLa and SiHa cells by RT-qPCR. (**C**-**E**) Validation of the reversal of the pro-proliferative effect of miR-4327 by TP53. (**C**) Changes in the viability of HeLa and SiHa cells. (**D**) Quantitative analysis of clone formation. (E) Representative images of clone formation. Scale bar showing 100 μm. (**F**) MiR-4327 targeting TP53 regulates the expression of proliferation and cell cycle-related proteins in cervical cancer. (**G**) Histogram of statistical analysis of data on proliferation and cell cycle protein levels in HeLa and SiHa cells. *p <0.05, **p <0.01, ***p <0.001, ****p <0.0001. #*p* <0.05, ##*p* <0.01, ###*p* <0.001, ####*p* <0.0001, compared with mimics NC+OE NC group

To explore the role of miR-4327 in cervical cancer cell proliferation via TP53, we co-transfected miR-4327 mimics and overexpressed TP53 (OE TP53) into HeLa and SiHa cells. Transfection efficiency was confirmed by RT-qPCR (Fig. 6A and B). CCK-8 assays revealed that TP53 overexpression reduced cell viability, while co-transfection with miR-4327 mimics and OE TP53 reversed this decline (Fig. 6C). Colony formation assays further showed that TP53 overexpression inhibited cell proliferation, but miR-4327 co-overexpression restored proliferation (Fig. 6D and E). To investigate the underlying mechanisms, we assessed the expression of tumor proliferation and cell cycle-related marker proteins using WB analysis (Fig. 6F and G). The results revealed that (Fig. 6F and G) lower levels of Ncad, Cyclin D1, and CDK4 in the OE-TP53 group compared to controls. In the co-transfection group, Ncad, Cyclin D1, and CDK4 were higher than in the OE-TP53 group. These findings suggest that TP53 overexpression counteracts miR-4327’s regulatory effects on cell proliferation and the cell cycle, supporting the miR-4327-TP53 targeting relationship.

## Discussion

The World Health Organization has launched a global strategy to eliminate cervical cancer by the end of this century through a three-tiered prevention system (Lin et al. 2023; Gultekin et al. 2020). While this offers hope, current treatments remain limited by immune suppression and toxicities such as radiation enteritis and myelosuppression (Ma et al. 2023; Corbeau et al. 2021). Newer therapies, including targeted therapy and immunotherapy, are gradually being integrated into clinical practice (Li et al. 2023). However, for patients with poor therapeutic responses, there is a need for optimized strategies to improve clinical outcomes. Cervical cancer progresses from persistent high-risk HPV infection to cervical intraepithelial neoplasia and eventually invasive cancer (Bowden et al. 2024). Understanding the molecular mechanisms underlying this progression is essential for early detection and personalized treatment. MiRNAs, particularly miR-4327, have emerged as key regulators of gene expression with potential diagnostic and therapeutic applications in cervical cancer (Causin et al. 2021). This study utilized the HeLa and SiHa cell lines, which have a well-established background of HPV infection, as in vitro models to systematically examine the cancer-promoting effects of miR-4327.

MiRNAs, small non-coding RNAs (19–25 nucleotides), have become crucial in molecular biology since the discovery of lin-4 in Caenorhabditis elegans by Lee et al. in 1993 (Lee et al. 1993). In cancer, dysregulated miRNA expression is linked to tumor initiation, progression, and metastasis (Abdelmonem et al. 2025; Zhang et al. 2025), functioning as either oncogenes or tumor suppressors. While miR-4327 remains underexplored, studies indicate its significant role in various cancers. It shows tissue-specific expression and functional variation, reflecting tumor microenvironment regulation. In esophageal cancer, altered miR-4327 expression is associated with tumor progression (Okuda et al. 2021). Additionally, miR-4327 levels are low in pancreatic ductal adenocarcinoma serum, suggesting its potential as a biomarker (Dong et al. 2016). In our study, miR-4327 was upregulated in cervical cancer tissues and its expression correlated with enhanced cell proliferation, migration, and invasion, confirming its oncogenic role and potential as a cervical cancer progression biomarker. Additionally, a systems biology study using HeLa cells demonstrated that miR-34a, miR-449a, and miR-16 regulate senescence, autophagy, apoptosis, and the G1/S checkpoint through both p53-dependent and -independent pathways (Gupta et al. 2022), highlighting the complex coordination of multiple cell fate decisions by miRNAs. This complements our finding that miR-4327, in contrast to these tumor-suppressive miRNAs, acts as an oncogene by directly targeting TP53 to promote proliferation.

The TP53 gene encodes the tumor suppressor protein p53, which regulates cell cycle, DNA repair, and apoptosis to maintain cellular homeostasis (Agarwal et al. 2015; Wang et al. 2022). P53 prevents tumorigenesis by inhibiting cell cycle progression, especially at the G1/S checkpoint, and inducing apoptosis (Charni et al. 2017). In the context of tumors, p53 suppresses the proliferation of damaged cells during the G1 phase, thereby preventing tumorigenesis (Vaddavalli and Schumacher 2022). However, TP53 mutations lead to loss of function, allowing uncontrolled cell proliferation and tumor growth (Hu et al. 2021). Mutant p53 also promotes metastasis via interactions with other signaling pathways (Su et al. 2024). Therefore, restoring the normal function of p53 or targeting specific mutant forms of p53 could provide therapeutic avenues to inhibit tumor proliferation and restore proper cell cycle regulation (Wan et al. 2020). Our study suggests that miR-4327 downregulates TP53 expression, disrupting these regulatory mechanisms and contributing to cervical cancer progression.

This study has several limitations that should be addressed in future research. Firstly, the xenograft tumor model used has certain limitations regarding its clinical relevance. Additionally, all functional assays and the xenograft model in this study were performed exclusively using HeLa and SiHa cells, as these two cell lines represent the most common subtypes of cervical cancer. However, we acknowledge that both cell lines are HPV‑positive. Therefore, our current findings may not be fully generalizable to other cervical cancer subtypes, particularly HPV‑negative lines or cervical cancers without HPV infection. Secondly, the relatively small sample size may affect the robustness and reliability of the results. Moreover, the downstream molecular mechanisms of the miR-4327/TP53 regulatory axis remains to be further elucidated. To enhance the study, we plan the following improvements: adopting an orthotopic tumor model with greater clinical relevance, expanding the sample size in animal experiments to increase statistical power, verifying the expression changes of key effector molecules downstream of TP53 (such as p21, Bax, etc.), and conducting an in-depth analysis of the downstream regulatory network of the miR-4327/TP53 signaling axis. These improvements will provide a more solid theoretical foundation for clinical translational research.

## Conclusion

Summarily, our study demonstrates that miR-4327 plays a crucial role in regulating cervical cancer cell proliferation. Furthermore, the target gene TP53, modulated by miR-4327, mediates alterations in the cervical cancer cell cycle, thereby promoting cellular proliferation. These findings offer valuable insights into the molecular mechanisms underlying cervical cancer proliferation and suggest potential strategies for the precise treatment of this malignancy.

## Supplementary Information

Below is the link to the electronic supplementary material.

Supplementary file1 (PDF 1207 KB)

Supplementary file2 (XLSX 1675 KB)

## Data Availability

Data supporting the results of this study can be obtained from the corresponding author upon reasonable request.
