# Supplementary material for: MiR-4327 targets TP53 to promote cervical cancer cell proliferation
Source: Funct Integr Genomics. 2026 Jul 11;26(1):187. doi: 10.1007/s10142-026-01934-9 (PMC13354660; doi:10.1007/s10142-026-01934-9)

Explanation

- For most blots, a single bright- field composite image is shown (marker visible, with chemiluminescent bands merged).
- For Fig. 5H (SiHa N- cadherin, ②) and Fig. 6G (HeLa CDK4 ①, TP53 ③, GAPDH ③) , two images are provided: bright-field (only for marker confirmation) and chemiluminescent (used for quantification).

Figure 5H

Lane 1	Lane 2	Lane 3	Lane 4
OE NC	OE	sh NC	sh

HeLa-Ncad-140kDa

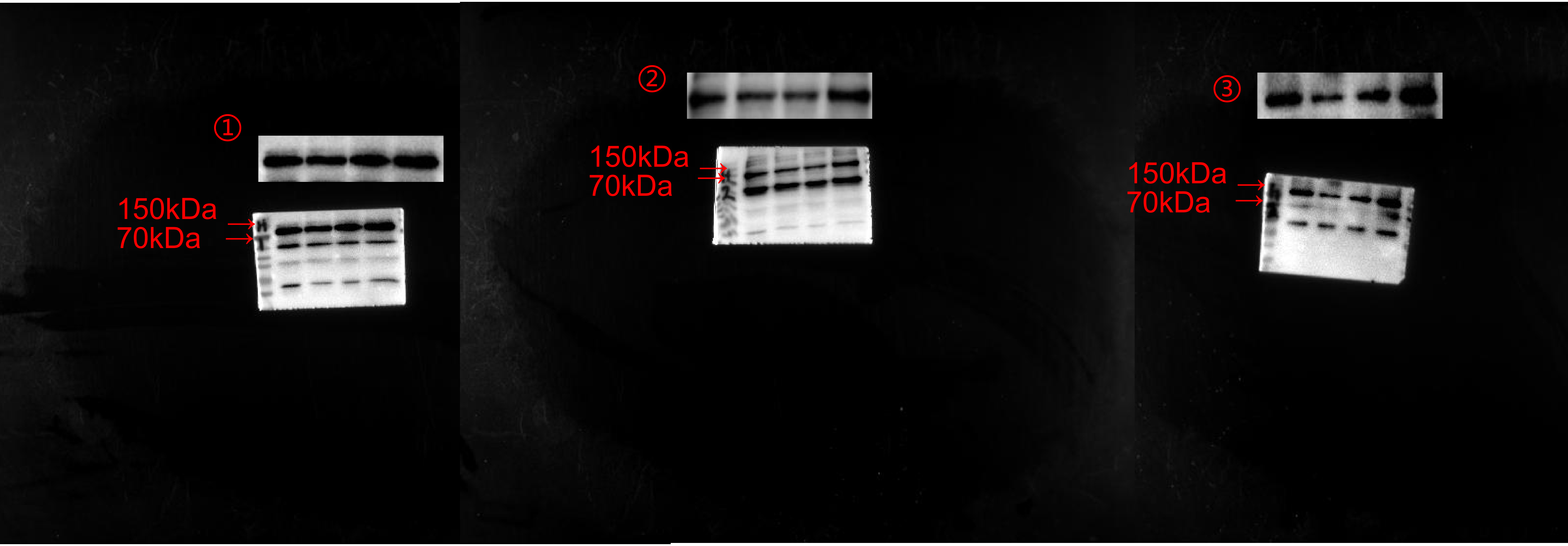

SiHa-Ncad-140kDa

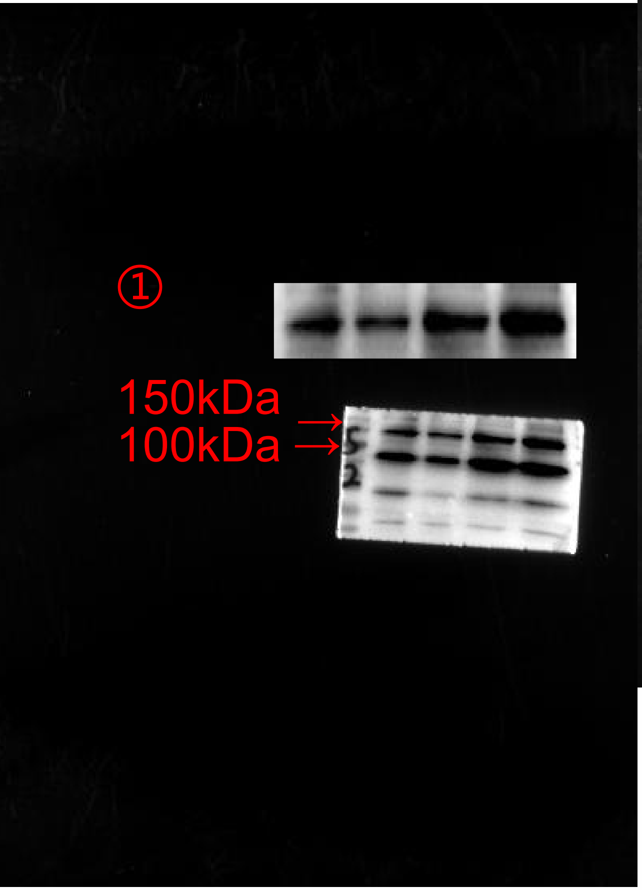

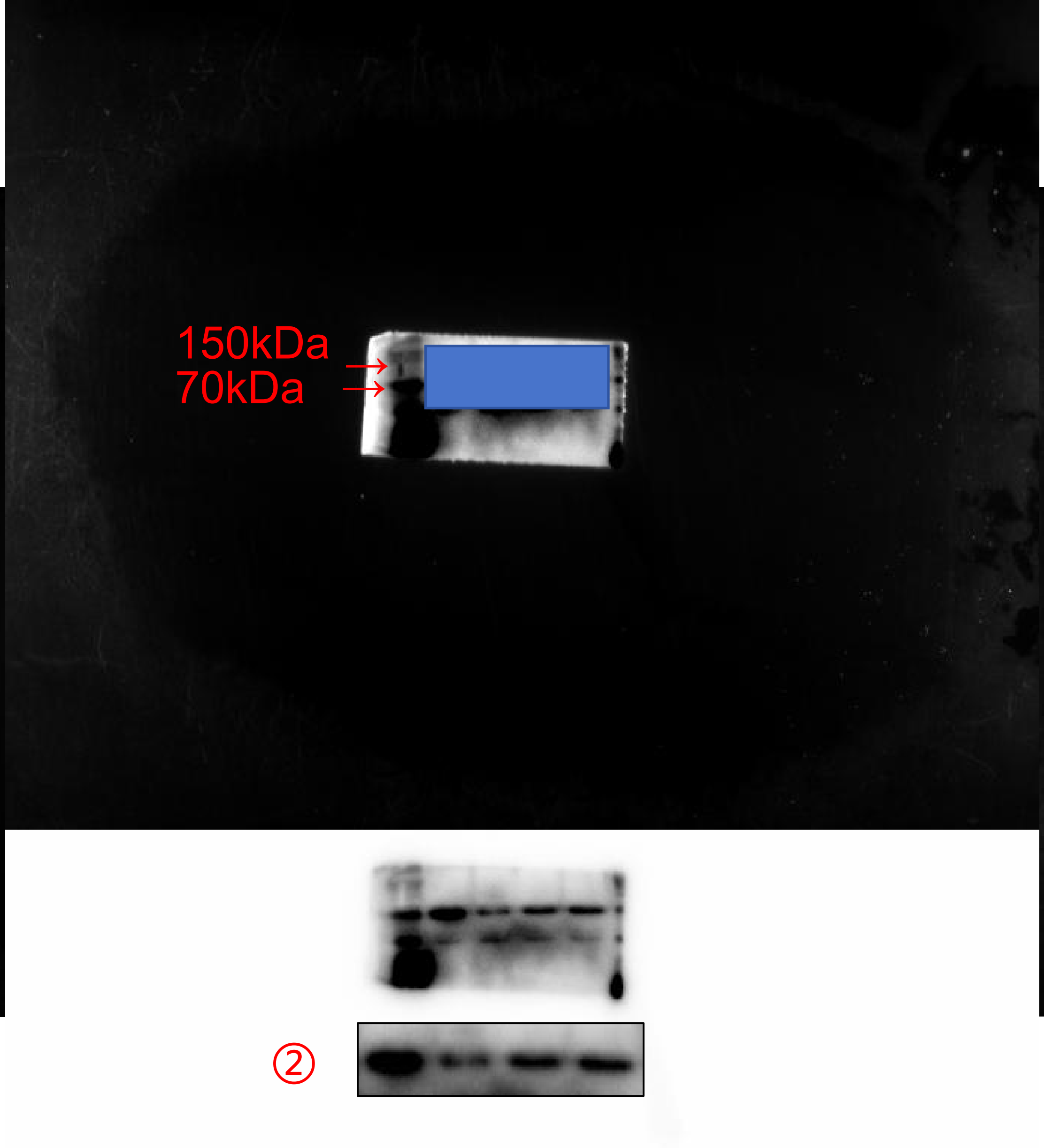

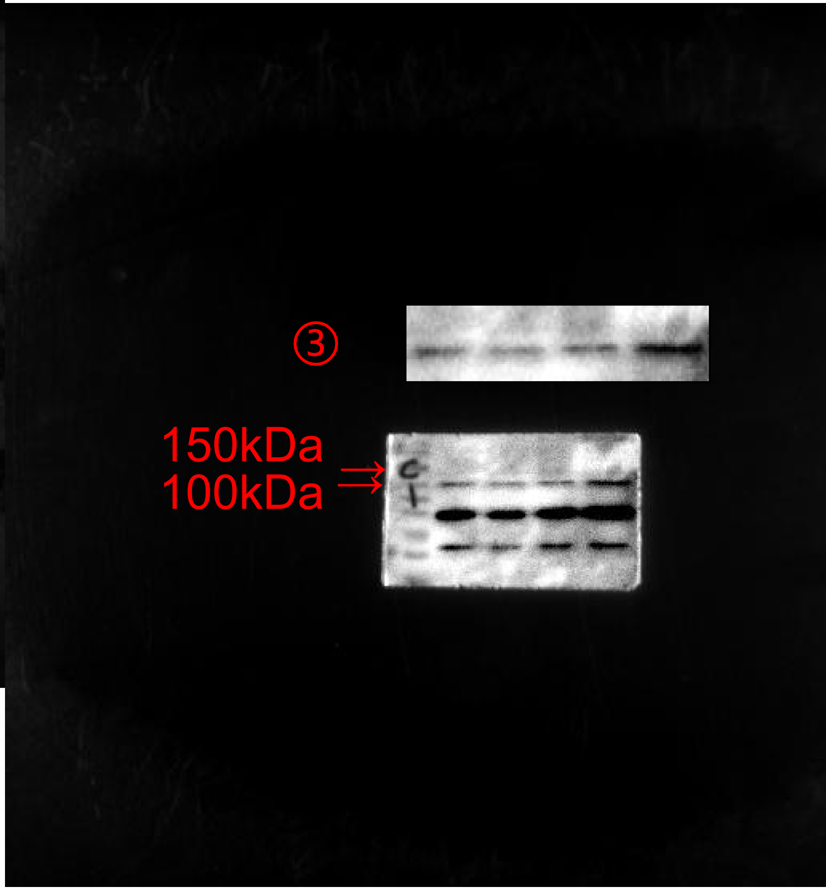

HeLa-CyclinD1-34kDa

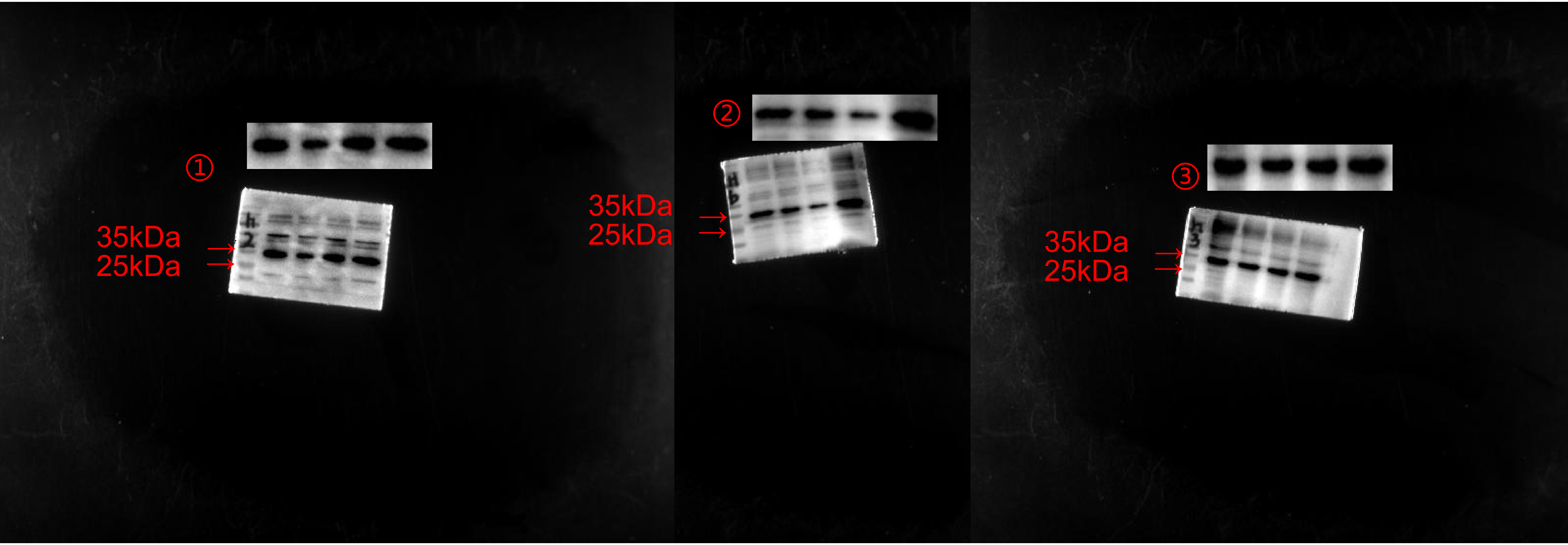

SiHa-CyclinD1-34kDa

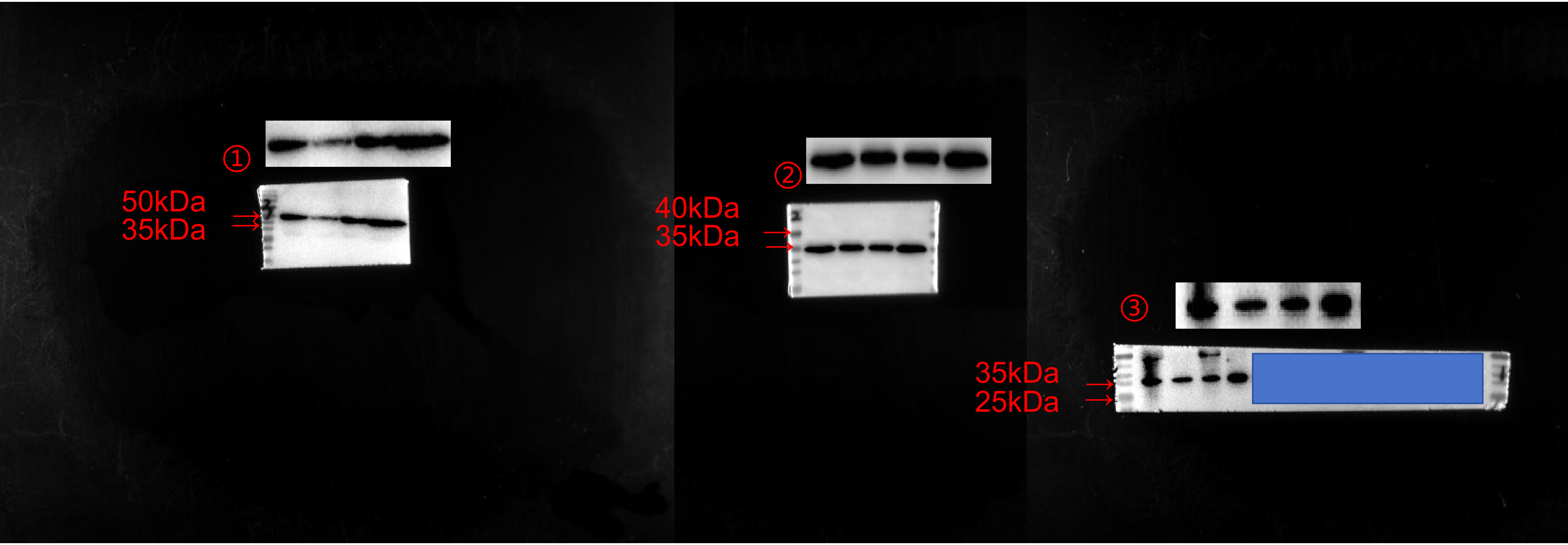

HeLa-CDK4-34kDa

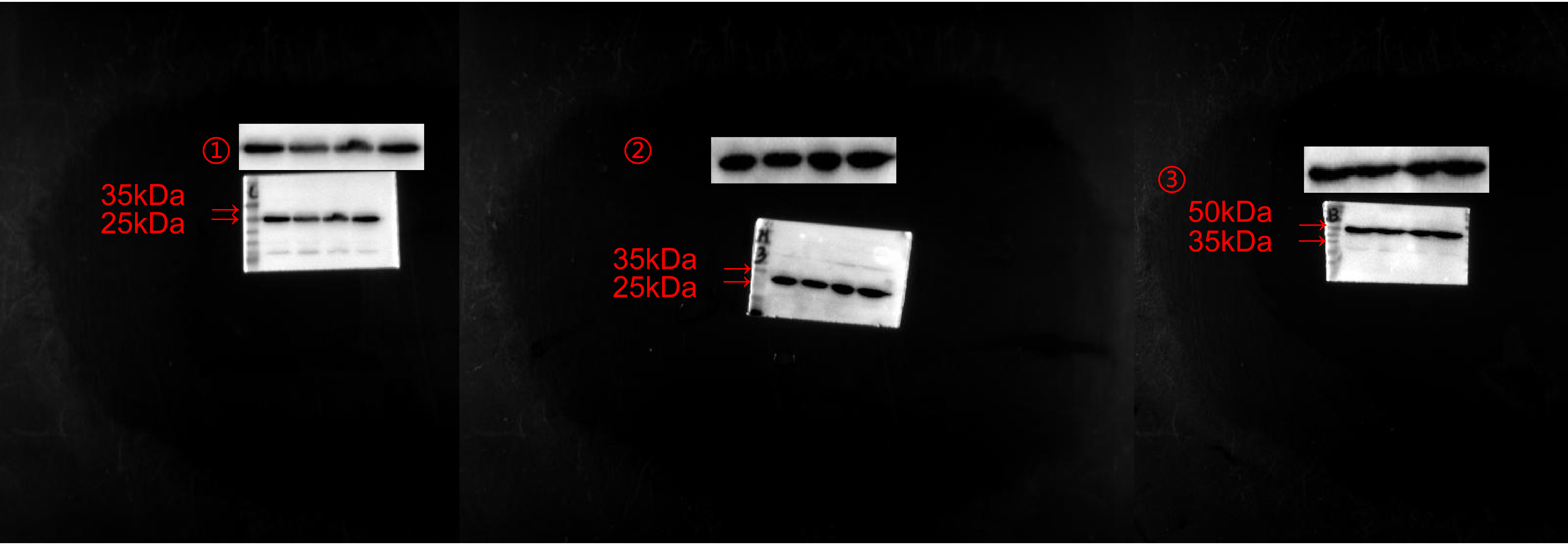

SiHa-CDK4-34kDa

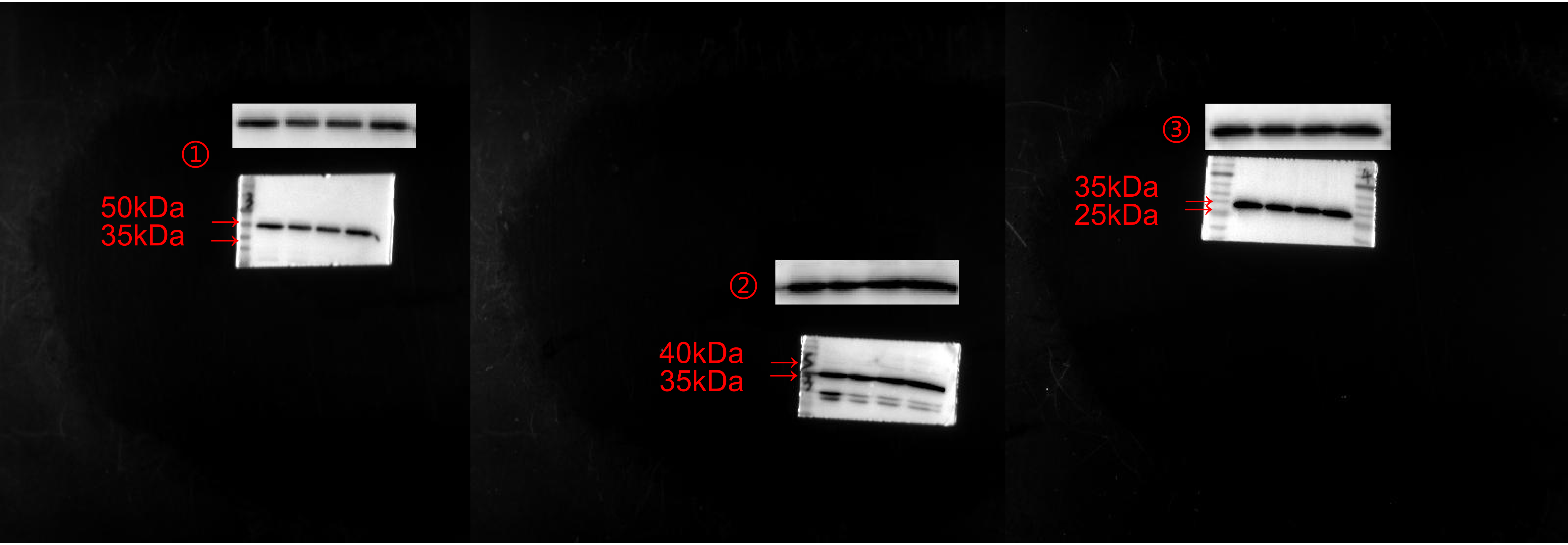

HeLa-TP53-53kDa

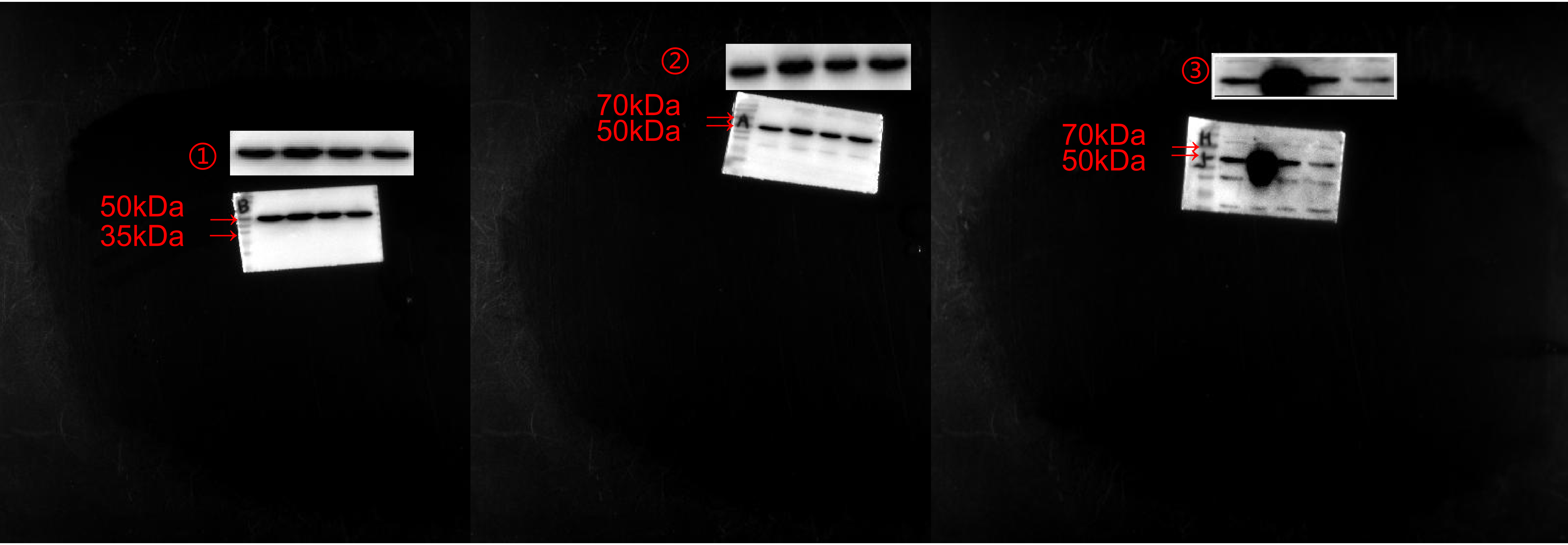

SiHa-TP53-53kDa

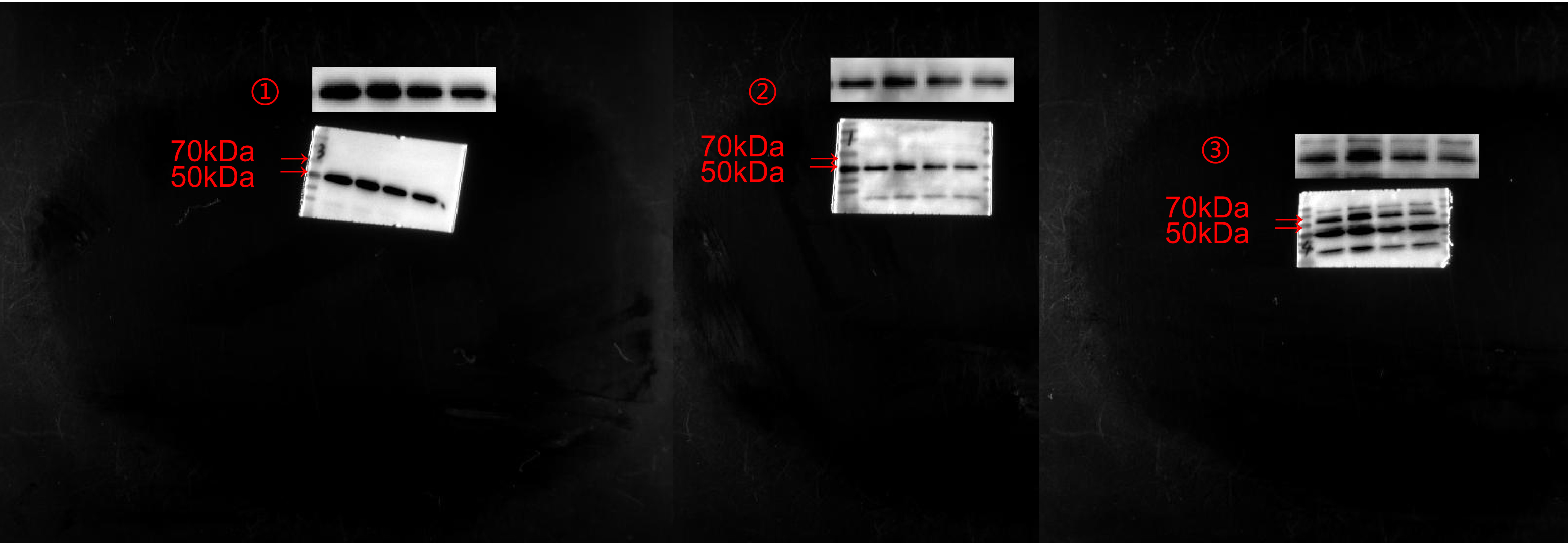

HeLa-GAPDH-37kDa

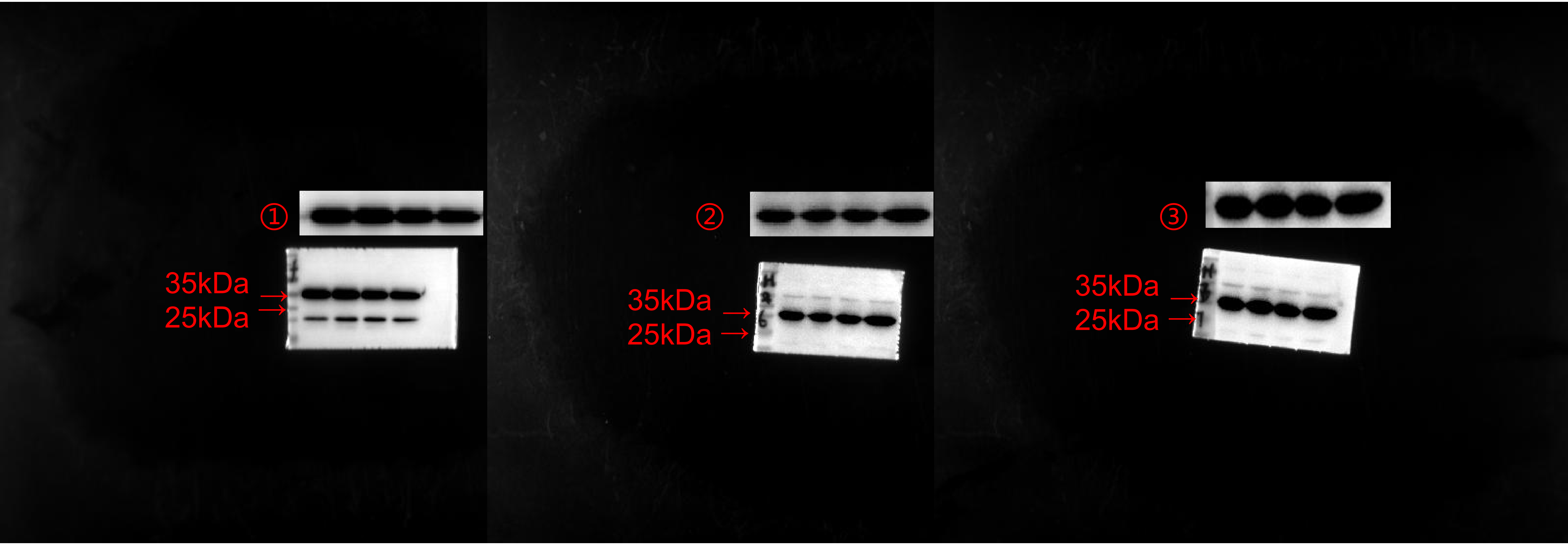

SiHa-GAPDH-37kDa

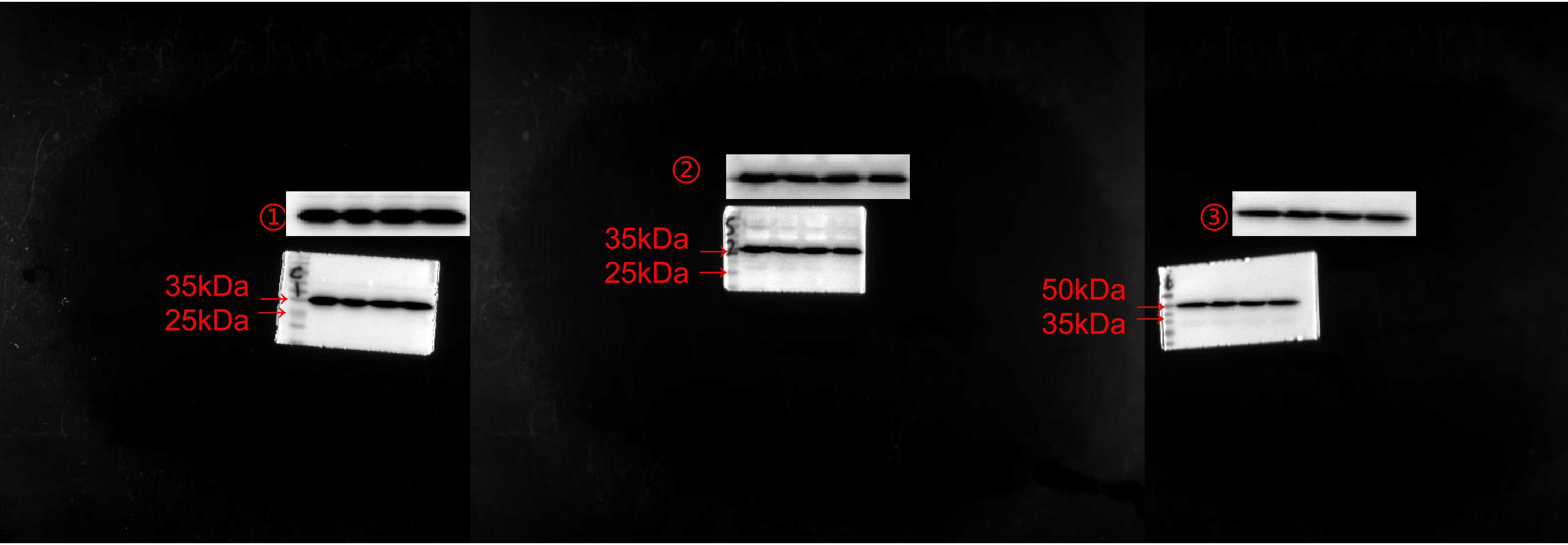

Figure 6G

Lane 1	Lane 2	Lane 3	Lane 4
mimics NC<br>+OE NC	miR-4327<br>mimics<br>+OE NC	mimics NC<br>+OE TP53<br>miR-4327	mimics<br>+OE TP53

HeLa-Ncad-140kDa

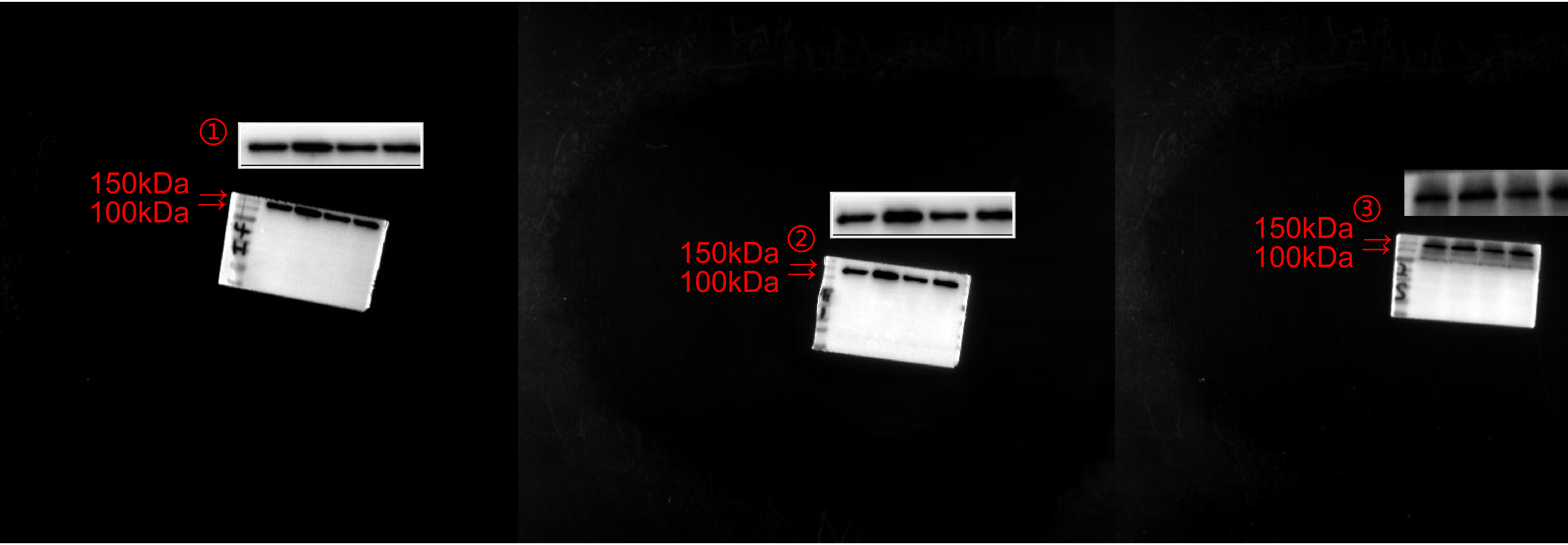

SiHa-Ncad-140kDa

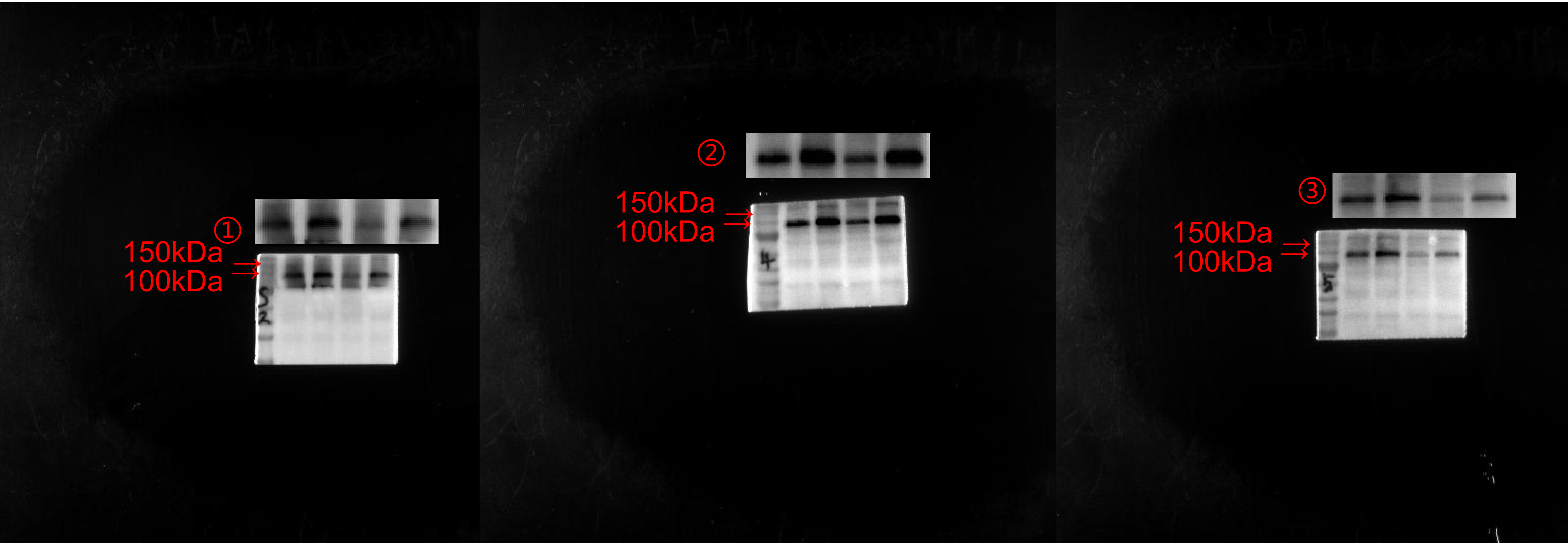

HeLa-CyclinD1-34kDa

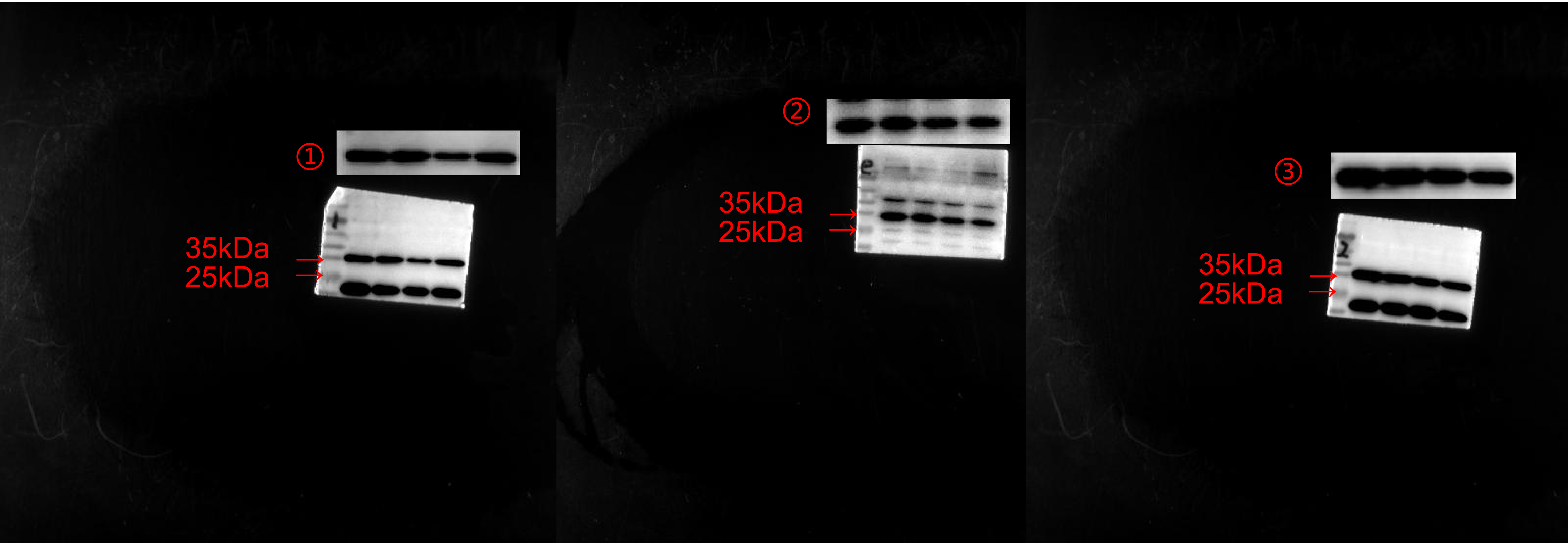

SiHa-CyclinD1-34kDa

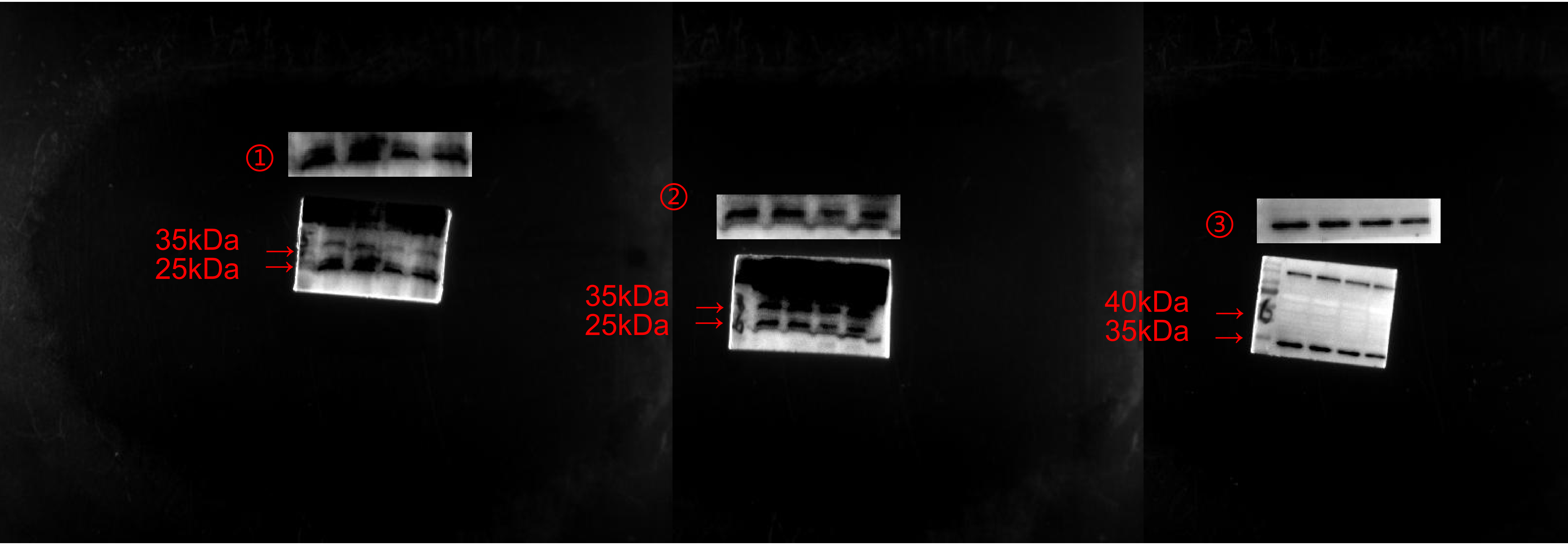

HeLa-CDK4-34kDa

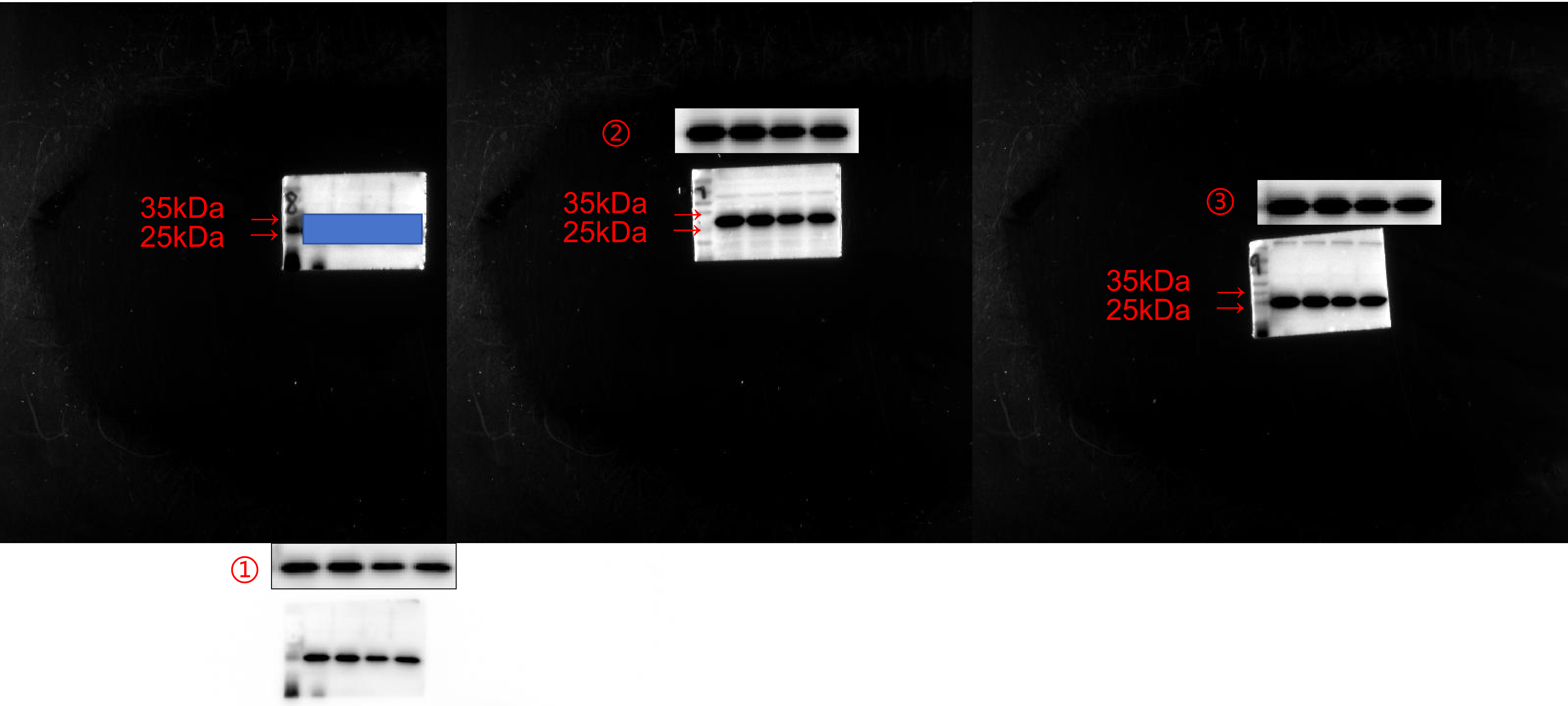

SiHa-CDK4-34kDa

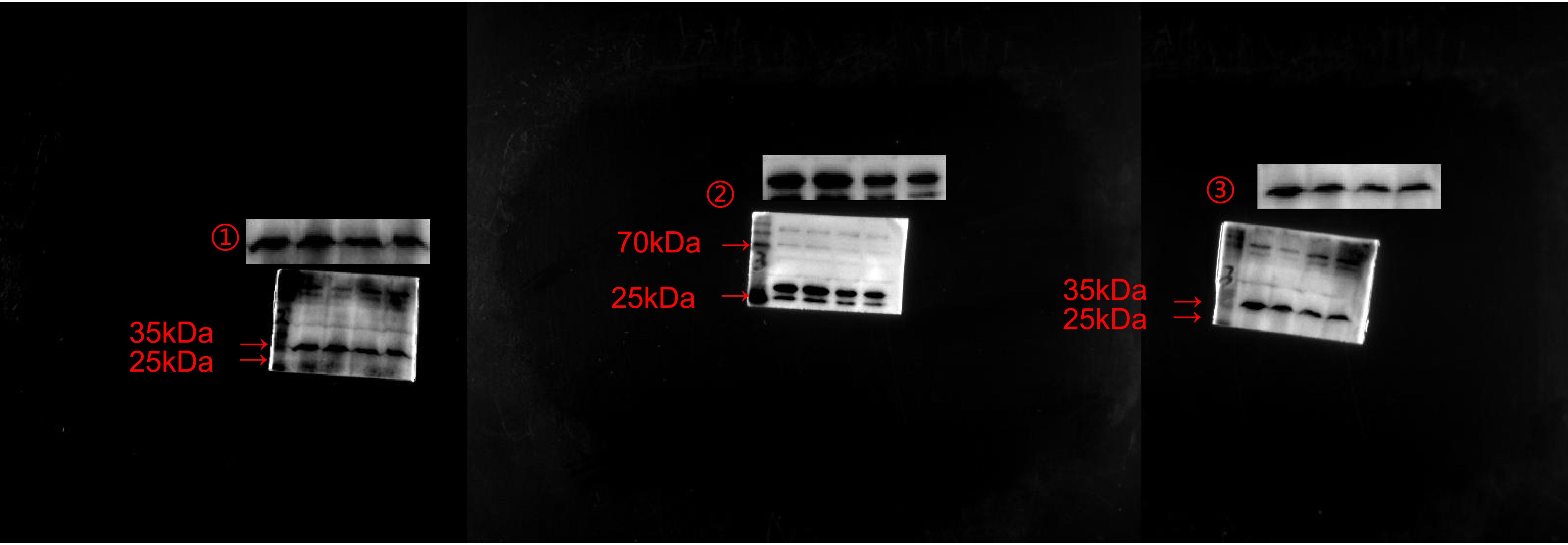

HeLa-TP53-53kDa

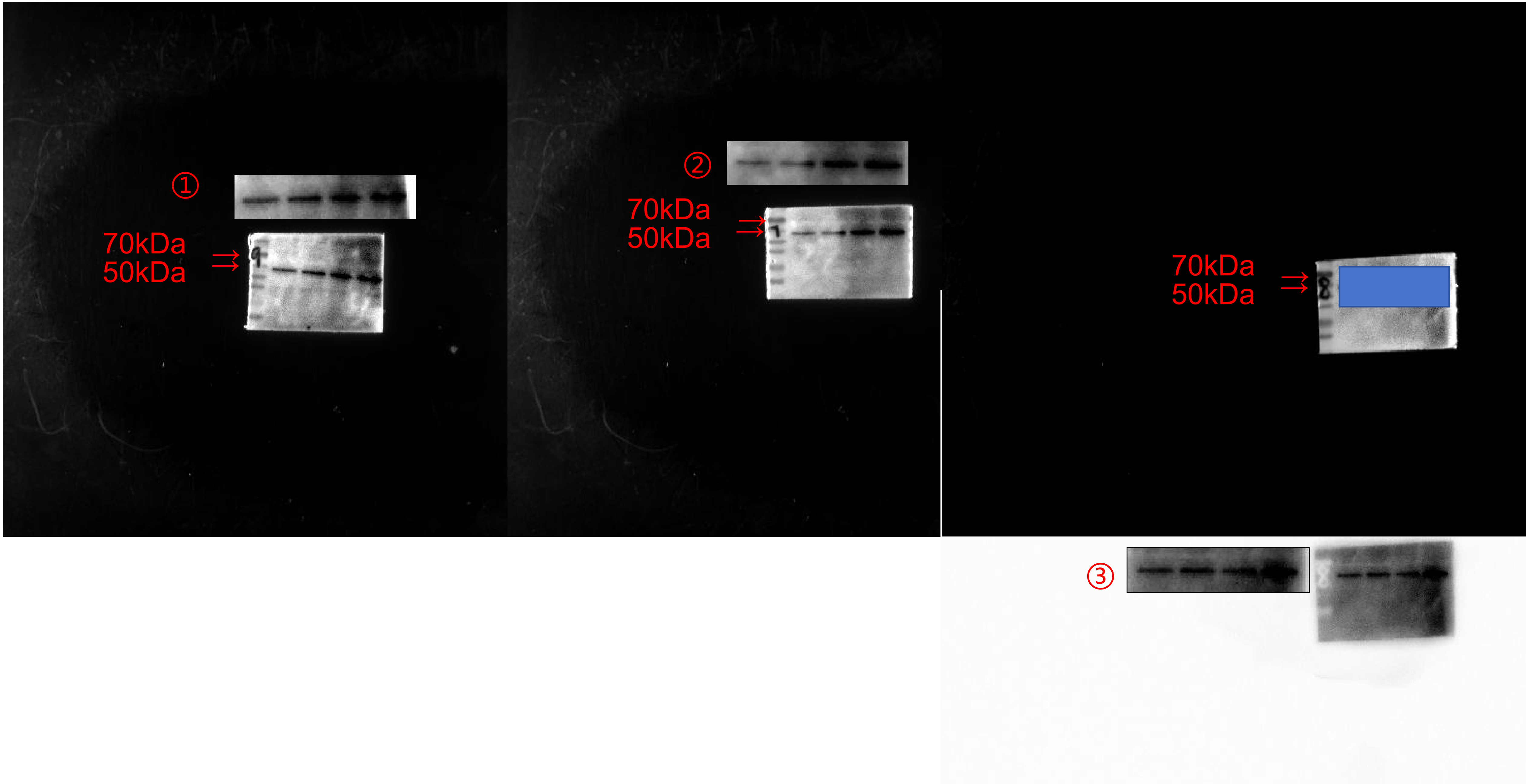

SiHa-TP53-53kDa

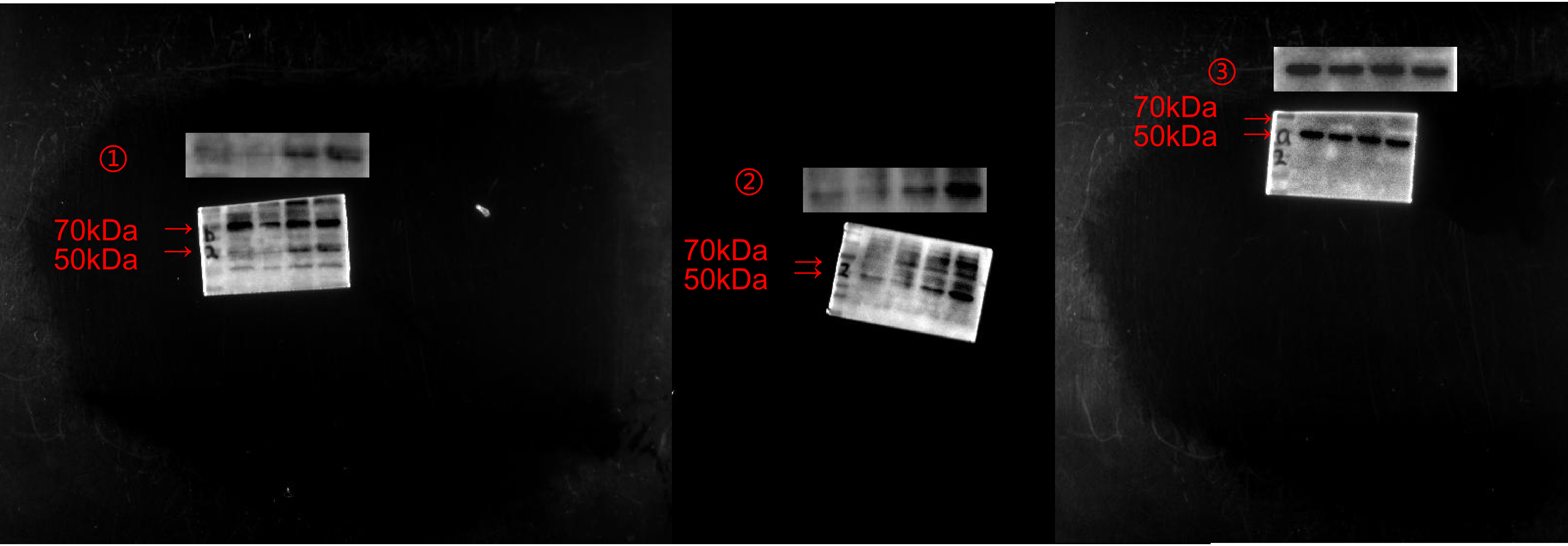

HeLa-GAPDH-37kDa

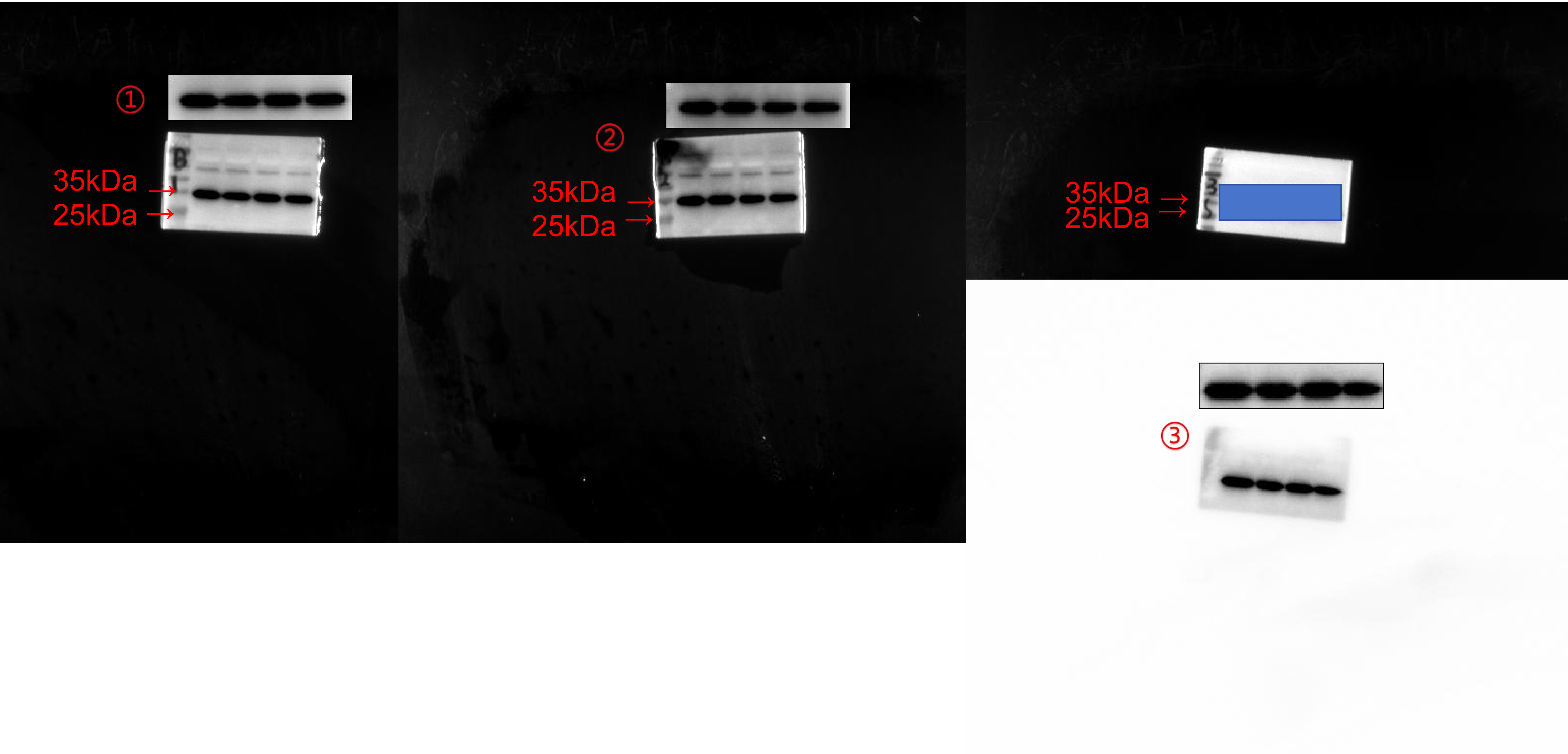

SiHa-GAPDH-37kDa

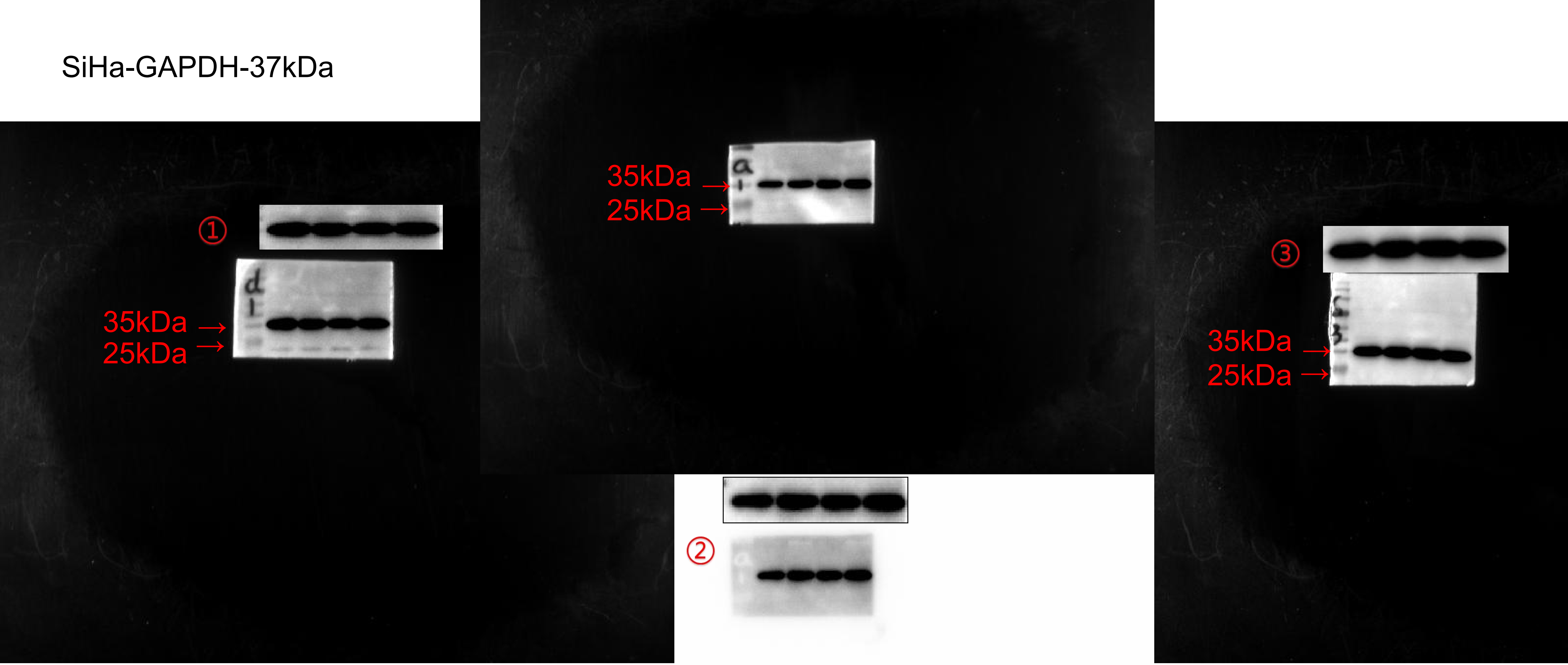

Supplement: Supplementary file 1 — Supplementary file1 (PDF 1207 KB) [file 10142_2026_1934_MOESM1_ESM.pdf]
